# Non-coding RNAs in the regulation of blood–brain barrier functions in central nervous system disorders

**DOI:** 10.1186/s12987-022-00317-z

**Published:** 2022-03-26

**Authors:** Ping Sun, Milton H. Hamblin, Ke-Jie Yin

**Affiliations:** 1 Department of Neurology, Pittsburgh Institute of Brain Disorders and Recovery, University of Pittsburgh School of Medicine, S514 BST, 200 Lothrop Street, Pittsburgh, PA 15213 USA; 2 Geriatric Research, Education and Clinical Center, Veterans Affairs Pittsburgh Healthcare System, Pittsburgh, PA 15261 USA; 3 Department of Pharmacology, Tulane University School of Medicine, 1430 Tulane Avenue, Mailcode 8683, New Orleans, LA 70112 USA

**Keywords:** microRNA, Long non-coding RNA, Circular RNA, Blood–brain barrier, Stroke, Traumatic brain injury, Spinal cord injury, Multiple sclerosis, Dementia, Brain tumor

## Abstract

The blood–brain barrier (BBB) is an essential component of the neurovascular unit that controls the exchanges of various biological substances between the blood and the brain. BBB damage is a common feature of different central nervous systems (CNS) disorders and plays a vital role in the pathogenesis of the diseases. Non-coding RNAs (ncRNAs), such as microRNAs (miRNAs), long non-coding RNA (lncRNAs), and circular RNAs (circRNAs), are important regulatory RNA molecules that are involved in almost all cellular processes in normal development and various diseases, including CNS diseases. Cumulative evidences have demonstrated ncRNA regulation of BBB functions in different CNS diseases. In this review, we have summarized the miRNAs, lncRNAs, and circRNAs that can be served as diagnostic and prognostic biomarkers for BBB injuries, and demonstrated the involvement and underlying mechanisms of ncRNAs in modulating BBB structure and function in various CNS diseases, including ischemic stroke, hemorrhagic stroke, traumatic brain injury (TBI), spinal cord injury (SCI), multiple sclerosis (MS), Alzheimer's disease (AD), vascular cognitive impairment and dementia (VCID), brain tumors, brain infections, diabetes, sepsis-associated encephalopathy (SAE), and others. We have also discussed the pharmaceutical drugs that can regulate BBB functions via ncRNAs-related signaling cascades in CNS disorders, along with the challenges, perspective, and therapeutic potential of ncRNA regulation of BBB functions in CNS diseases.

## Overview of non-coding RNAs

Non-coding RNAs (ncRNAs) refer to the RNA molecules that do not have the protein-coding potential, which account for the majority of RNAs and make up about 98–99% of all mammalian genomes generated RNAs [1, 2]. Although non-coding RNAs do not possess the ability to translate to proteins, increasing evidence demonstrated that they actively interact with nucleic acids or other molecules to function as vital regulatory molecules on almost all cellular processes in normal development and various diseases, including the central nervous system (CNS) disorders [1, 3–5]. In addition, one ncRNA is able to interact with one or more target molecules in different cellular signaling pathways, making the ncRNA-mediated regulation network even more complicated. For endogenous ncRNAs, besides structural ncRNAs such as ribosomal RNA (rRNAs) and transfer RNAs (tRNAs), ncRNAs can be broadly divided into small ncRNAs (<200 nucleotides) and long ncRNAs (>200 nucleotides). Small ncRNAs can be even subdivided into microRNAs [6], small interfering siRNA (siRNAs) [7], piwi-interacting RNAs (piRNAs) [8], small nuclear RNAs (snRNAs) [9], small nucleolar RNAs (snoRNAs), [10] and others [11]. Long ncRNAs include long intronic ncRNAs, antisense RNAs (asRNAs), promoter-associated long RNAs (PALRs), promoter upstream transcripts (PROMPTs), stable excised intron RNAs, and long stress-induced non-coding transcripts (LSINCTs) [12]. Numerous ncRNAs exhibit regulatory functions on the blood–brain barrier (BBB) during CNS diseases, which mainly include microRNAs, long non-coding RNAs, and circular RNAs.

MicroRNAs (miRNAs, miRs) are a class of single-stranded non-coding RNAs with an average length of 22 nucleotides [13]. They can inhibit protein expression mainly by complementary binding to the messenger RNAs (mRNA) of target genes at the 3’ untranslated region (3’ UTR) to induce mRNA degradation or translational repression [6]. miRNAs are very stable, can be secreted to various biofluids by different types of cells, and be transported by Argonaute protein or exosomes, which can protect their degradation from RNases, suggesting them as potential biomarkers during disease biogenesis [5–7]. Also, miRNAs have been shown to be able to target hundreds of target genes by each of them and can modulate more than one-third of all human genes [5, 14, 15], suggesting their vast involvement and complex regulatory functions.

Long non-coding RNA (lncRNAs) are defined as a heterogeneous class of mRNA-like transcripts that are longer than 200 nucleotides but without protein-coding ability [3, 16]. Similar to mRNAs, their transcription is also involved in RNA Polymerase II (RNAPII), and most of the lncRNAs are polyadenylated [12]. In general, lncRNAs lack the open reading frame, show tissue-specific expression profile, and exhibit low levels compared to the protein-coding counterparts [3, 12]. The structure features of the lncRNAs play a key role in modulating the affinity of DNA, RNA, and proteins, in the assembly and regulation of multi-molecular complexes [3]. It has been shown that lncRNAs can regulate gene expression through different mechanisms, including modulation of gene promotor or transcription factor activity and splicing machinery, recruitment of transcriptional activator, increasing mRNAs stability or inducing their decay, acting as architectural components in the assembly of protein complexes, or served as competing endogenous RNAs (ceRNAs) to regulate the functions of miRNAs [3, 16, 17].

Unlike linear ncRNAs (e.g., microRNAs or lncRNAs), circular RNA (circRNA) is a class of covalently closed single-stranded long ncRNA that the 5’ and 3’ termini are connected by back-splicing circularization of exons of pre-mRNAs and forms a continuous loop [18, 19]. Due to the unique circular structure, circRNAs are more resistant to be degraded by RNases, thereby more stable than linear ncRNAs, and can be served as potential biomarkers in some diseases. Also, it has been suggested that circRNAs exhibit tissue-specific and cell-specific expression properties [19]. The growing interest in this relatively new type of ncRNAs gradually reveals the biological functions of circRNAs, including miRNA sponges, protein sponges or decoys, enhancer of protein functions, protein scaffolding and recruitment, and serving as templates for translation under certain circumstances [19, 20].

## Overview of the blood–brain barrier and neurovascular unit in the central nervous system

The blood–brain barrier (BBB) plays a vital role in controlling the influx and efflux of various biological substances between the brain and the blood to maintain the metabolic activity and function of the brain [21]. Serval excellent articles have systematically reviewed the structure, cellular components, and biological functions of the BBB [21–23]. At the cellular level, BBB is formed by brain microvascular endothelial cells (BMECs), astrocytes end-feet and pericytes, and cell-cell adhesion molecules (mainly tight junctions, TJs) tightly seal a monolayer of brain endothelial cells in the capillary microvasculature to form a highly selective diffusion barrier. The diffusion barrier can selectively prevent the passive exchange of solutes and neurotoxic molecules, regulate the trafficking of macromolecules, ions, amino acids, peptides, and signaling molecules, and the entry of leukocytes [24–27] between the blood and the brain [23, 28].

The junction complex of BBB consists of tight junctions (TJs) and adherens junctions (AJs) [21]. The TJs mainly comprised of three types of integral membrane proteins, including claudins (e.g., claudin-1, claudin-2, claudin-3, claudin-5, claudin-11, etc.), occludin, junction adhesion molecules (JAMs) (e.g., JAM-A, JAM-B, JAM-C, JAM-4, JAM-5, etc.), and cytoplasmic accessory proteins (e.g., zonula occludens-1 (ZO-1), ZO-2, ZO-3, cingulin, etc.), which form the cytoplasmic bridge connecting the TJs to the cell cytoskeleton [22, 23]. At the BBB, it is broadly consented that claudin-5 is the most abundant and dominant claudin in endothelial cells, which plays a key role in maintaining BBB integrity, and its dysfunction has been associated with various neurological disorders [29–32]. The AJs mainly consist of cadherins, which join the actin cytoskeleton to form adhesive contacts between cells through intermediary submembrane proteins, catenins (e.g., β-catenin, γ-catenin) [21–23]. Beta-catenin and γ-catenin connect the cytoplasmic domains of cadherins to the cell cytoskeleton via α-catenin [21–23].

Pericytes are also crucial constituents of the BBB and brain capillary, and they are abundantly expressed in the CNS. They share a basement membrane with endothelial cells and contact with endothelium through N-cadherin and connexins [21, 33]. Pericytes play a crucial role in maintaining BBB integrity, facilitating angiogenesis, and stabilizing the structure of microvasculature [21, 23, 33, 34]. Astrocytes are also essential components of BBB and may play a decisive role in the induction of BBB characteristics and maintaining the barrier function of brain endothelial cells. Astrocytic end-feet has also been suggested as crucial checkpoints of brain metabolism [21, 23, 35]. The neurovascular unit consists of all the major cellular components of the brain, including brain endothelial cells, vascular smooth muscle cells (VSMC), pericytes, astrocytes, neurons, microglia, and perivascular cells [36]. The communication between the cells of the neurovascular unit is vital and responsible for regulating blood flow and controlling the exchange of substances across the BBB [37].

## Non-coding RNAs as diagnostic and prognostic biomarkers for BBB damage in the CNS disorders

Emerging evidence has suggested the diagnostic and therapeutic values of circulating non-coding RNAs in human diseases, and some excellent articles have reviewed microRNAs, long non-coding RNAs, or circular RNAs as promising non-invasive biomarkers for numerous CNS disorders. For example, microRNAs can be served as diagnostic and prognostic biomarkers of ischemic stroke [5, 38], hemorrhagic stroke [39], traumatic brain injury (TBI) [40], spinal cord injury (SCI) [41], glioma [42], multiple sclerosis (MS) [43], Alzheimer’s disease (AD) [44], vascular cognitive impairment and dementia (VCID) [45], and others. LncRNAs have also been suggested as diagnostic and therapeutic biomarkers for stroke [46], TBI [47], SCI [48], glioma [49], MS [50], AD [51], and other diseases. In addition, several circular RNAs (e.g., circ_101222) have been suggested as potential biomarkers for neurological diseases [52]. Although these articles summarized the general potential of non-coding RNAs as diagnostic and prognostic biomarkers for CNS diseases, their regulation on BBB was not specified. Thus, our current paper reviews the non-coding RNAs able to regulate BBB functions and possess the potential to be served as promising biomarkers of CNS diseases.

### microRNAs

Several microRNAs have been suggested as potential biomarkers for BBB disruption in ischemic stroke, hemorrhagic stroke, TBI, SCI, MS, breast cancer brain metastasis, AD and sepsis-associated encephalopathy (SAE). Wang et al. described that elevated miR-29b levels in white blood cells related to impaired BBB functions, which also negatively associated with NIHSS (National Institute of Health stroke scale) scores and brain infarct volume in ischemic stroke, and could potentially predict stroke outcomes as a novel circulating biomarker [53]. Zhang et al. showed that the significantly increased plasma miR-503 positively correlated with plasma von Willebrand Factor (vWF, an indicator of EC dysfunction) in ischemic stroke patients [54]. miR-503 was also an independent risk factor for ischemic stroke by logistic analysis [54]. Additionally, miR-503 was able to regulate BBB damage, brain edema, and cerebral circulation by modulating endothelial monolayer permeability, oxidative stress, and cell apoptosis, suggesting miR-503 as a promising biomarker for ischemic stroke with BBB regulation functions [54]. miR-130a was significantly elevated in the serum of intracerebral hemorrhage (ICH) patients and rat ICH models, associated with clinical outcomes in patients with deep hematoma, and was an independent indicator positively associated with perihematomal edema (PHE) volume within the first three days after the onset of ICH in patients [55]. Besides, brain edema, BBB permeability, and neurological deficit scores can be significantly alleviated by miR-130a inhibitors, making miR-130a a useful early biomarker for monitoring post-ICH PHE and predicting disease prognosis [55]. miR-21 has been proposed by several groups to be a promising biomarker for both TBI patients and preclinical animal models, and its upregulation upon TBI inhibits endothelial apoptosis, enhances junctional proteins expression, and targets angiogenic factors critical to BBB maintenance [40]. miR-155 has been suggested as a potential biomarker in spinal cord ischemic injury, as miR-155 exhibited upregulated expression in neurons and endothelial cells of spinal cord ischemic injury mice, but showed lower miR-155 expression in mice that escaped paralysis [56]. miR-155 deletion also reduced central cord edema and improved blood-spinal cord barrier (BSCB) integrity with reduced incidence of spinal cord paralysis [56]. miR-155 has also been suggested to serve as potential prognostic biomarkers for relapsing-remitting multiple sclerosis (RRMS) [57]. Different from SCI, miR-155 exhibited reduced expression in blood samples of RRMS patients. More interestingly, downregulated miR-155 significantly correlated with patients’ expanded disability status scale (EDSS) and upregulated surface receptors and cytotoxic proteins that were crucial for migration through the BBB, such as intracellular adhesion molecule 1 (ICAM-1) and integrin subunit beta 2 (ITGB-2) in CD8^+^ T cells [57]. In breast cancer brain metastasis, miR-105 is specifically expressed and secreted by the breast cancer cell. Cancer-secreted miR-105 is also significantly associated with metastatic progression in breast cancer patients by downregulating tight junction protein zonula occludens 1 (ZO-1), destroying the endothelial monolayer barrier function, and inducing vascular permeability. These results suggested miR-105 as an early diagnostic blood-borne marker as well as a therapeutic target for breast cancer metastasis [58]. In AD, miR-181a might be a biomarker for the progression of the disease, as miR-181a declined synchronously with the accumulation of beta-amyloid (Aβ) in APP/PS1 mouse, suggesting a reverse correlation between miR-181a levels and AD development [59]. The decline of miR-181a is also correlated with Aβ accumulation-induced pericyte apoptosis and BBB breakdown in APP/PS1 mice [59]. Furthermore, plasma miR-370-3p has been suggested as an attractive biomarker candidate in SAE [60]. Sepsis-induced BBB breakdown is considered a significant cause of SAE. Increased miR-370-3p expression was specially observed in the brain but not in other organs in SAE mouse models [60]. Plasma miR-370-3p was also specifically increased and highly sensitive for early detection in SAE in patients or animal models with BBB permeability defect, neuroinflammation, and brain apoptosis, suggesting plasma miR-370-3p a unique biomarker for SAE [60].

### Long non-coding RNAs

In ischemic stroke, lncRNA XIST has been proposed to be a potential biomarker for predicting the prognosis of acute cerebral ischemia and a therapeutic target for stroke patients. LncRNA XIST exhibited increased expression during the late stages (seven days later) after the onset of ischemic stroke, and the serum levels of lncRNA XIST were significantly negatively correlated with the severity of neurological impairments in the ischemic stroke patients [61]. Silencing of lncRNA XIST significantly impaired angiogenesis by decreasing the endothelial migration and tube formation, exacerbated cerebral vascular injury by markedly reducing the expressions of KLF4 and tight junction proteins claudin-5 and ZO-1, and evidently increased the vascular permeability by upregulating the expression of E-selectin, vascular cell adhesion protein 1 (VCAM-1), ICAM-1, and p-NF-kB [61]. Another lncRNA, named lncRNA associated with breast cancer brain metastasis (BCBM) (Lnc-BM), has also been suggested as a prognostic biomarker of the progression of brain metastasis in breast cancer patients [62]. Lnc-BM is overexpressed in breast cancer tissue and is upregulated specifically in brain metastatic cells, but not in lung or bone metastatic cells [62]. High Lnc-BM expression was negatively correlated with recurrence-free survival in breast cancer patients, and high Lnc-BM expression in the primary tumor predicts an increased risk for brain metastasis [62]. In addition, elevated Lnc-BM expression promotes BCBM, while depletion of Lnc-BM effectively suppresses BCBM. Moreover, Lnc-BM enhances the STAT3 (signal transducer and activator of transcription 3)-dependent expression of ICAM1 and CCL2 (C-C chemokine ligand 2, monocyte chemoattractant protein-1, MCP-1), which mediated vascular invasion and recruitment of macrophages in the brain, respectively, to increase the cancer cell migration through BBB and to exacerbate BCBM [62].

### Circular RNAs

Circular RNAs are abundantly expressed in eukaryotes, spatiotemporal specific, highly conserved, and less susceptible to degradation by RNA exonucleases; thus, they have recently been proposed to be novel biomarkers for CNS disorders, including ischemic stroke. However, there are relatively fewer publications of this type of non-coding RNAs that possess the ability to regulate BBB functions in different CNS disorders and serve as biomarkers. One circular RNA, named circRNA DLGAP4 (circDLGAP4), has recently been suggested as a promising biomarker for diagnosing and evaluating the degree of cerebral damage caused by acute ischemic insults [63]. CircDLGAP4 levels were significantly decreased in the plasma of acute ischemic stroke patients and a mouse model of ischemic stroke, and upregulation of circDLGAP4 expression significantly inhibited miR-143 activity and resulting in improved neurological deficits, decreased infarct areas, and attenuated blood–brain barrier extravasation along with ameliorated the downregulation of tight junction proteins, including claudin-5, occludin, and ZO-1 in the preclinical ischemic stroke models [63]. These findings suggested cirDLGAP4 in the plasma as a novel biomarker and therapeutic target in acute cerebrovascular protection after ischemic stroke [63].

## Non-coding RNAs regulate BBB/BSCB functions in CNS disorders

**Fig. 1:**
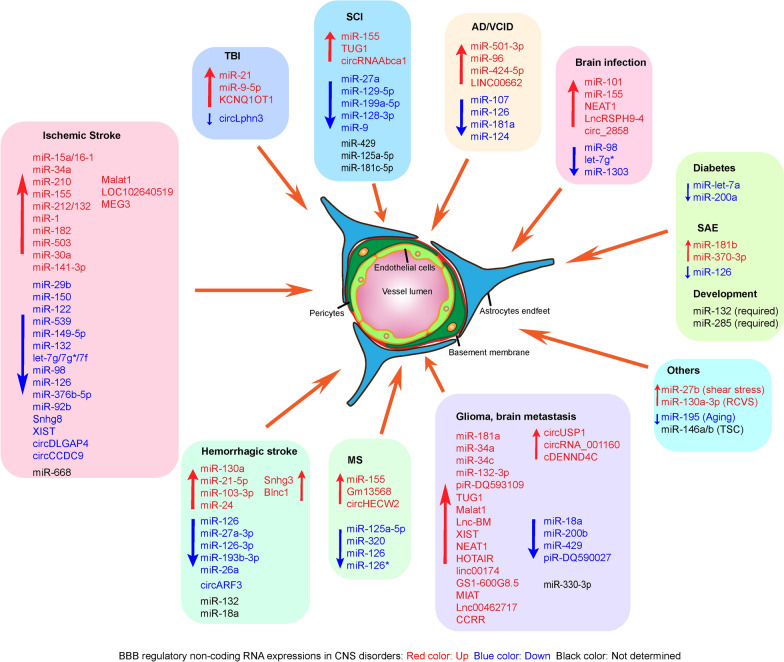
Altered expression profiles of BBB regulatory miRNAs, lncRNAs, and circRNAs in CNS disorders. During CNS disorders, such as ischemic stroke, hemorrhagic stroke, traumatic brain injury (TBI), spinal cord injury (SCI), glioma and brain metastasis, multiple sclerosis (MS), Alzheimer's disease (AD), vascular cognitive impairment and dementia (VCID), brain infections, diabetes, sepsis-associated encephalopathy (SAE), and others, the structure and the function of BBB has been compromised along with dysregulated non-coding RNA expressions. Modulating the expressional levels of miRNAs, lncRNAs, and circRNAs is able to effectively regulate the BBB damage and recovery during these CNS disorders. “↑”, upregulated non-coding RNA levels, and “↓” downregulated non-coding RNA levels in CNS disorders. *RCVS* reversible cerebral vasoconstriction syndrome; *TSC* tuberous sclerosis complex

Accumulating evidence has demonstrated the extensive involvement (Fig. 1) and regulatory mechanisms of different non-coding RNAs (microRNAs (Table 1), lncRNAs (Table 2), and circular RNAs (Table 3) in BBB/BSCB injuries and repairs in various CNS disorders. Herein, we summarized functional significance and molecular mechanisms of different non-coding RNAs in CNS disorders, including ischemic stroke and hemorrhagic stroke, TBI, SCI, brain tumors (glioma, glioblastoma, and brain metastasis), MS, dementia, brain infections, diabetes, SAE and others.

**Table 1:** microRNAs in the regulation of BBB functions in different CNS disorders

CNS disorders	Year	miRNAs	miRNA levels in disease	Study materials	Main regulatory effects on BBB	Main mechanisms	Refs.
Ischemic stroke	2010	miR-15a	Up	Mice, CECs	Downregulation of miR-15a protects BBB and reduces cerebral infarction	Upregulation of PPARϭ reduces ischemia-induced miR-15a, resulting in posttranscriptional inhibition of bcl-2	[64]
	2015	miR-29b	Down	Mice	miR-29b reduces infarct volume, edema, BBB disruption	miR-29b overexpression directly targets Aquaporin-4	[53]
	2016	miR-34a	Up	bEnd.3	miR-34a increases BBB permeability and disrupts ZO-1, impairs mitochondrial oxidative phosphorylation, reduces ATP production	miR-34a directly targets cytochrome c (CYC C)	[67]
	2016	miR-150	Down	Rats, BMECs	miR-150 increases BBB permeability and decreases cell survival	miR-150 could directly regulate the angiopoietin receptor Tie-2 and downregulate claudin-5 expression	[70]
	2016	miR-122	Down	Rats	Systematic miR-122 administration decreases neurological deficits, brain infarction and maintains BBB integrity	miR-122 mimic downregulates direct target genes (e.g., VCAM-1, iNOS, PLA2G2A) and indirect target genes (e.g., ALOX5, ITGA2b, TIMP-3, IL-1b, IL-2, MMP-8)	[71]
	2017	miR-210	Up	Neonatal rats	miR-210 exacerbates cerebral edema and BBB leakage	miR-210 directly targets tight junction protein occludin and catenin	[72]
	2018	miR-21		Rats	miR-21 reduces ischemic hemisphere volume, cerebral infarct volume, BBB permeability	miR-21 directly targets MAP2K3, and decreases p38, MAP2K3, iNOS, MMP-9	[73]
	2018	miR-539	Down	Rats, BMECs	miR-539 alleviates BBB Disruption	miR-539 suppresses MMP-9 expression	[74]
	2018	miR-155	Not detected	hBMECs	miR-155 inhibition improves endothelial monolayer integrity	miR-155 directly targets claudin-1, and miR-155 inhibition increases claudin-1 and ZO-1	[75]
	2018	miR-149-5p	Down	Rats, mBVPs	miR-149-5p decreases pericyte migration, attenuates BBB permeability, and improves the outcomes	miR-149-5p negatively regulates S1PR2, increases N-cadherin expression and decreases pericyte migration	[77]
Ischemic stroke/TBI	2019	miR-212/132	Up	Mice, hBMECs	miR-212/132 overexpression decreases barrier properties and reduces EC migration	miR-212/132 overexpression decreases claudin-1, JAM3, and TJAP1	[222]
Ischemic stroke	2019	miR-1	Up	Rats	miR-1 inhibition improves neurological deficits, reduces infarction volume, brain edema, and BBB permeability	N/A	[78]
	2019	miR-132	Down	Rats	miR-132 decreases the infarct volume, reduces brain edema, preserves the integrity of BBB, and improves neurological functions	miR-132 suppresses MMP-9 mRNA and decreases the degradation of tight junction proteins VE-cadherin and β-Catenin	[79]
	2019	miR-34a	Up	Mice, CECs	miR-34a disrupts ZO-1 and induces BBB permeability	miR-34a reduces the expression of CYC C	[68]
	2020	miR-15a/16–1	Up	Mice, mBMECs	EC miR-15a/16-1 cKO reduces brain infarcts, BBB leakage, and decreases infiltration of peripheral immune cells	miR-15a/16-1 directly targets claudin-5. EC-selective miR-15a/16-1 deletion enhances claudin-5 levels and reduces M1-type microglia/macrophage infiltration	[65]
	2020	let-7g*/miR-98	Down	Mice	Let-7g* and miR-98 reduce proinflammatory cytokines, let-7g* reduces BBB leakage and improves vascular perfusion function	Both miRs directly target CCL2 and CCL5 cytokines to regulate the cerebral microvessel thickness and perfusion	[80]
	2020	let-7g	Down	Mice, BMVEC	Let-7g attenuates inflammation, reduces BBB permeability	Let-7g limits neutrophils infiltration, microglia activation, and neuronal death	[81]
	2020	miR-126-3p/5p	Down	Mice	miR-126-3p or -5p reduces brain infarct volume, edema volume, and IgG leakage to attenuate BBB disruption	miR-126-3p and -5p downregulates proinflammatory cytokines and adhesion molecules, and attenuates the decrease of ZO-1 and occludin	[82]
	2020	miR-34a	Up	Mice, CECs	Knockout of miR-34a reduces BBB permeability and improves stroke outcomes	miR-34a directly targets cytochrome c and alleviates disruption of tight junctions	[69]
	2020	miR-98	Down	Mice	miR-98 attenuates BBB permeability, reduces infarct size, and improves locomotor impairment	miR-98 ameliorates the infiltration of proinflammatory Ly6CHI leukocytes and microglial polarization	[83]
	2020	miR-155	Up	Mice	miR-155 KO reduces cerebral infarct volume, hemorrhagic burden and improves neurological deficits	miR-155 deficiency reduces hemorrhagic transformation via the effects on BBB integrity and permeability	[76]
	2020	miR-668	Not detected	Rats	miR-668 inhibition reduces the infarct area, BBB permeability, and neurological score	miR-668 inhibition attenuates the levels of NLRP3, ZO-1, and occludin proteins	[84]
	2020	miR-182	Up	Mice, bEnd.3	miR-182 KO attenuates infarct volume, BBB permeability	miR-182 directly targets mTOR and FOXO1 to exacerbate apoptosis and the loss of TJ proteins ZO-1 and occludin	[85]
	2020	miR-503	Up	Mice, hBMECs	miR-503 downregulation attenuates the neurological injury, BBB damage, brain edema, CBF reduction	miR-503 regulates EC permeability, cell apoptosis, NO, and ROS generation via PI3K/Akt/eNOS pathway or bcl-2/cleaved caspase-3 pathway	[54]
	2021	Let-7f	Down	bEnd.3	Let-7f increases cell viability, inhibits apoptosis, reduces endothelial permeability	Let-7f targets HMGA2 (high mobility group AT-hook 2) to regulate cell viability, apoptosis, TJ proteins (ZO-1 and occludin), endothelial permeability, and BBB function	[86]
	2021	miR-30a	Up	Mice, bEnd.3	miR-30a inhibition reduces infarct volume, ameliorates neurological deficits, decreases BBB permeability, prevents degradation of TJ proteins	miR-30a directly targets the zinc transporter ZnT4 and regulates ZnT4/zinc signaling pathway	[87]
	2021	miR-376b-5p	Down	Mice	miR-376b-5p improves the BBB permeability, relieves brain edema and decreases infarct area	miR-376b-5p targets SOX7 to mediate the Wnt/β-catenin signaling	[88]
	2021	miR-149-5p	Down	Rats	miR-149-5p decreases neurological defects, tissue damage, and brain water content, and increases BBB integrity	miR-149-5p decreases the level of inflammatory cytokines (TNF-α and IL-6), MMP-2, and MMP-9	[89]
	2021	miR-92b	Down	Rats, BMECs	miR-92b improves neurological function and protects the BBB integrity	miR-92b targets and negatively modulates NOX4 expression to regulate the viability and permeability of ECs	[90]
	2021	miR-141-3p	Up	Mice	miR-141-3p inhibition reduces infarct injury might be due to improved BBB integrity	Not specified	[91]
Hemorrhagic stroke	2016	miR-130a	Up	Mice, BMECs	miR-130a inhibition reduces brain edema, BBB permeability and improves neurological scores	miR-130a level decreases caveolin-1 and increases MMP-2/9	[55]
	2017	miR-126	Down	Rat, BMECs	miR-126-3p inhibits neutrophil infiltration, microglial activation, and neuronal apoptosis	miR-126-3p suppresses phosphoinositide-3-kinase regulatory subunit 2 (PIK3R2), maintains Akt activation, inhibits apoptosis	[106]
	2016	miR-132	Not detected	Mice	miR-132 enhances the expression of claudin-5 and ZO-1, alleviates BBB permeability, neuro-deficits, brain edema, inflammatory response, and neuronal death	miR-132 reduces the level of AChE and enhances the protective effect of ACh on the brain	[223]
	2018	miR-27a-3p	Down	Rats, BMECs	miR-27a-3p alleviates behavioral deficits, brain edema, vascular leakage, and leukocyte infiltration	miR-27a-3p suppresses aquaporin-11 (AQP11)	[109]
	2019	miR-126-3p	Down	Rats, BMECs	miR-126-3p inhibition impairs BMECs barrier permeability and upregulates VCAM-1	miR-126-3p direct targets VCAM-1	[107]
	2019	miR-21-5p	Up	Rats, HEK293T	miR‐21‐5p inhibition reduces the neurological defects, cognitive impairment, alleviates BBB permeability, neuronal apoptosis, and neuroinflammation	miR‐21‐5p direct targets DUSP8 and deactivates the p‐ERK/HO‐1 pathway	[103]
	2020	miR-126a-3p	Not detected	Rats, MSCs	miR-126 facilitates the differentiation of MSCs into vascular ECs. miR-126-MSC transplantation reduces the neurological severity score, brain water content, and BBB permeability	miR-126-modified MSC alleviates the cell apoptosis, diminishes PAR-1 and MMP-9, enhances ZO-1 and claudin-5	[108]
	2020	miR-193b-3p	Down	Mice, BMSCs	miR-193b-3p mitigates the neurological behavioral impairment, brain edema, neurodegeneration, and BBB permeability	miR-193b-3p suppresses the expression and activity of HDAC3, upregulating the acetylation of NF-κB p65	[110]
	2021	miR-18a	Not detected	Rats, BMECs	miR-18a enhances BBB permeability, brain edema, and impairs neurological functions	miR-18a direct targets RUNX1, and decreases RUNX1, occludin, and ZO-1	[224]
	2021	miR-103-3p	Up	Mice, bEnd.3	miR-103-3p inhibition reduces BBB permeability, preserves microvascular integrity, and improves long-term neurobehavioral function	miR-103-3p directly interacts with Caveolin-1 and decreases ZO-1 and occludin	[104]
	2021	miR-24	Up	Rats	miR-24 downregulation improves the learning and memory abilities and improves BBB integrity	miR-24 targets HMOX1, miR-24 inhibition increases SOD, decreases MDA, MPO, IL-6, and TNF-α	[105]
	2021	miR-26a	Down	Rats	Overexpressing miR-26a in SHED (stem cells from human exfoliated deciduous teeth) attenuates brain water content and BBB permeability in ICH rats	miR-26a direct targets connective tissue growth factor (CTGF), which has close associations to cell apoptosis	[111]
TBI	2014	miR-21	Up	Rats	Upregulation of miR-21 improves long-term neurological function, alleviates brain edema, lesion volume, and BBB leakage	miR-21 promotes the expression of VEGF and the Ang-1/Tie-2 axis. miR-21 also inhibits PTEN and activates Akt signaling	[116]
	2015	miR-21	Up	Rats	Upregulation of miR-21 improves neurological outcomes, alleviates secondary BBB damage and loss of TJ proteins occludin and claudin-5	miR-21 activates the Ang-1/Tie-2 axis, promotes the expression of TJ proteins occludin and claudin-5 to stabilize BBB	[117]
	2016	miR-21-5p	UP	BMECs	miR-21-5p alleviates BBB leakage, upregulates occludin and claudin-5 expression after scratch injury	miR-21-5p suppresses inflammatory cytokines and NF-kB signaling, inhibits cellular apoptosis, and promotes the Ang-1/Tie-2 axis	[118]
	2019	miR-9-5p	Up	Rats, BMECs	Upregulation of miRNA-9-5p improves neurological recovery, alleviates apoptosis, neuroinflammation, and BBB damage	miRNA-9-5p targets Ptch-1, activates the Hedgehog/AKT/GSK3β pathway, and inhibits NF-κB/MMP-9 pathway	[119]
SCI	2015	miR-27a	Down	Rats	miR-27a attenuates BSCB leakage after I/R injury, and inhibition of miR-27a exacerbates BSCB leakage	miR-27a attenuates TLR4 activation and inflammatory damage to the BSCB	[124]
	2017	miR-129-5p	Down	Mice	miR-129-5p preserves motor function, prevents BSCB leakage and water content	miR-129-5p targets the high-mobility group box-1 (HMGB1), and decreases toll-like receptor (TLR)-3, interleukin (IL)-1β and TNF-α levels	[125]
	2018	miR-155	Up	Mice	miR-155 deletion reduces central cord edema, improves BSCB integrity, and reduces the incidence of spinal cord paralysis	miR-15a targets Mfsd2a in EC and motorneurons, exacerbates gray matter damage and EC permeability	[56]
	2018	miR-199a-5p	Down	Rats	miR-199a-5p improves the limb motor function and reduces BSCB leakage	miR-199a-5p negatively regulates endothelin converting enzyme 1 (ECE1), and regulates caspase-9, Bcl-2, p-JNK, p-ERK signaling	[126]
	2020	miR-128-3p	Down	Rats	miR-128-3p ameliorates BSCB leakage and proinflammatory cytokine release, including IL-6, TNF-a, and IL-1β	miR-128-3p directly targets specificity protein 1 (SP1) to alleviate neuroinflammation and cell apoptosis	[127]
	2020	miR-429	Not detected	hCMEC/D3, spinal cord astrocytes	miR-429 inhibition increases TJ protein ZO-1, occludin, and claudin-5 to reduce BSCB permeability	miR-429 negatively regulates KLF6 to mediate TJ protein expression and BSCB permeability	[131]
	2020	miR-125a-5p	Not detected	SCMECs	miRNA-125a-5p reduces permeability and EC death rates	miRNA-125a-5p upregulates ZO-1, occludin, and VE-cadherin, and against hypoxia-induced apoptosis	[129]
	2021	miR-155	Not detected	Mice, bEnd.3	miR-155 promotes EndoMT and aggravates BSCB disruption, and inhibits the expression of TJ proteins (ZO-1, occludin, claudin1, 2, and 5)	miR-155 activates the NF-κB pathway by suppressing SOCS6-induced p65 degradation	[130]
	2021	miR-9	Down	Rats	miR-9 improves neurological function, reduces BSCB disruption and leakage	miR-9 reduces MAP2K3 and Notch2, reduces the release of IL-6 and IL-1β, and cleaved-caspase 3	[128]
	2021	miR-181c-5p	Not detected	rBMECs, spinal cord astrocytes	NHO-1 reduces the BSCB disruption, and levels of miR-181c-5p. miR-181c-5p increases the BSCB permeability under hypoxia	miR-181c-5p directly targets SOX5 (sex determining region Y-box protein 5)	[132]
Glioma/brain metastasis	2014	miR-181a	Up	GECs	miR-181a overexpression increases BTB permeability	miR-181a direct targets KLF6, and downregulates TJ protein ZO-1, occludin, and claudin-5	[138]
	2014	miR-34c	Up	GECs	miR-34c overexpression impairs BTB integrity and increases BTB permeability	miR-34c regulated BTB permeability via MAZ-mediated expression changes of ZO-1, occludin, and claudin-5	[139]
	2015	miR-18a	Down	GECs	miR-18a overexpression increases the BTB permeability	miR-18a directly bound to myocyte enhancer factor 2D (MEF2D), and MEF2D could directly bind to KLF4 promoter to regulate ZO-1, claudin-5, and occludin	[140]
	2015	miR-18a	Down	GECs	miR-18a overexpression impairs the integrity and increases the permeability of BTB	miR-18a increases the permeability of BTB via RUNX1-mediated down-regulation of TJ proteins ZO-1, occludin, and claudin-5	[141]
	2015	miR-34a	Up	GECs	miR-34a overexpression increases the permeability of BTB	miR-34a regulates TJ proteins and BTB function by targeting protein kinase Cε	[142]
	2016	miR-200b	Down	GECs	miR-200b overexpression inhibits BTB leakage	miR-200b targets RhoA and ROCKII to regulate stress fiber formation and TJ disassembly	[143]
	2017	miR-330-3p	Decreased by EMAP-II	GECs	Endothelial monocyte-activating polypeptide-II (EMAP-II) decreases miR-330-3p, which can decrease BTB permeability	EMAP-II increases BTB permeability through inhibiting miR-330-3p, which negatively regulates PKC-α, and suppresses ZO-1, occludin, and claudin-5	[144]
	2018	piR-DQ590027/miR17HG	Down	GECs	piR-DQ590027 overexpression increases the permeability of glioma conditioned normal BBB	piR-DQ590027 bound to and negatively regulates MIR17HG, which bound separately to miR-153 and miR-377 and negatively regulates FOXR2/ZO-1, occludin, and claudin-5 expression	[145]
	2018	miR-429	Down	GECs	miR-429 overexpression decreases the expression of ZO-1, occludin, and claudin-5, and reduces the distribution continuity	miR-429 directly targets ZO-1, occludin, and p70S6K to increase BBB permeability	[146]
	2018	piRNA-DQ593109	Up	GECs	Downregulation of PIWIL1 or piR-DQ593109 increases BTB permeability	Downregulating PIWIL1 and piR-DQ593109 increases BTB permeability through the MEG3/miR-330-5p/RUNX3/ZO-1, occludin, claudin-5 axis	[147]
	2019	miR-132-3p	Up	rats, GECs	miR-132-3p increases BTB permeability	miR-132-3p contributes to the increased permeability of BTB by targeting PTEN/PI3K/PKB/Src/Cav-1 axis	[148]
Multiple sclerosis (MS)	2013	miR-125a-5p	Down	hCMEC/D3, human fetal astrocytes, patients’ brains	miR-125a-5p overexpression increases brain EC barrier function	miR-125a-5p increases VE-cadherin and ZO-1, and reduces TNF-α-mediated ICAM-1 expression and monocyte transmigration through the EC barrier	[168]
	2014	miR-155	Up	mice/brain tissue from MS patients	miR-155 knockout reduces CNS extravasation, and endogenous miR-155 partially prevents the cytokine-induced increase in permeability	miR-155 targets cell–cell complex molecules (annexin-2 and claudin-1), and targets cell-to-extracellular matrix interactions (dedicator of cytokinesis 1 (DOCK-1) and syntenin-1 (SDCBP))	[172]
	2015	miR-320a	Down	Patients’ blood samples	MMP-9 is increased, and miR-320a is decreased in B lymphocytes of MS patients during disease relapse compared to remission	miR-320a inhibition upregulates intracellular MMP-9 protein in B cells and increases extracellular secretion	[169]
	2016	miR-155	Up	hCMEC/D3	Endothelial miR-155 upregulates the leukocytes extravasation across the inflamed BBB	miR-155 positively regulates VCAM1 and ICAM1 levels	[173]
	2017	miR-126&-126*	Down	hCMEC/D3	Reduction of endothelial miR-126 and miR-126* enhances firm monocyte and T cell adhesion to ECs	miR-126* and miR-126 downregulation increase E-selectin and VCAM1, respectively. miR-126 overexpression reduces VCAM1 and CCL2 expression	[170]
	2021	miR-155 from CD8^+^ T cells	Down	Relapsing–Remitting MS patients’ samples	miR-155 is downregulated, while ICAM-1 and ITGB2 are upregulated	Downregulation of miR-155 correlates with upregulation of surface receptors and cytotoxic proteins in CD8^+^T cells	[57]
AD/VCID	2016	miR-107	Down in AD	HBMECs	Overexpression of miR-107 abrogates Abeta-induced disruption of BBB and endothelial cell dysfunction	miR-107 direct targets and downregulates endophilin-1, and regulates BBB permeability and the expression of ZO-1, occludin, and claudin-5	[177]
	2018	miR-501-3p	Up in VCID	Mice	miR-501-3p inhibition attenuates BBB disruption within the white matter and ameliorates memory deficits	miR-501-3p directly targets human ZO-1 and downregulates trans-endothelial electric resistance	[182]
	2018	miR-126	Down in VCID	Mice	EC-miR-126 cKO exacerbates cognitive impairment, decreases CBF, myelin and axon density, increases inflammation, water channel, and glymphatic impairment	miR-126 knockout increases endothelial MMP-9 and TLR4 inflammatory factor, downregulates AQP-4, and delays penetration and clearance of CSF into the brain via paravascular pathways	[181]
	2018	miR-96	Up	Mice, hBMECs	High levels of GM-CSF downregulates ZO-1 and facilitates the infiltration of peripheral monocytes across the BBB	miR-96 targets ETS transcription factor ERG to downregulate ZO-1 expression	[179]
	2019	miR-181a	Down in AD	APP/PS1 mice, mBVPs	miR-181a overexpression ameliorates cognitive deficits and amyloid plaque deposition	miR-181a attenuates pericyte apoptosis and BBB breakdown by suppressing FOXO1	[59]
	2019	miR-124	Down in AD	APP/PS1 mice	miR-124 rescues BBB breakdown, promotes angiogenesis, reduces Aβ deposition, and alleviates learning and memory deficit	miR-124 targets and suppresses C1ql3 to upregulate expression of ZO-1 to enhance BBB integrity	[178]
	2019	miR-424-5p	Up in AD	hCMEC/D3	miR-424-5p silencing decreases BBB permeability	miR-424-5p silencing upregulates TJ proteins ZO-1 and occludin by targeting Endophilin-1	[180]
Brain infection	2013	miR-101	Up	BMVECs	HIV-1 Tat C increases the expression of miR-101 and increases EC permeability	miR-101 directly targets VE-cadherin, and VE-cadherin levels govern the expression of claudin-5	[185]
	2015	miR-98 and let-7g*	Down	Mice, BMECs	Overexpression of let-7 and miR-98 reduces leukocyte adhesion to and migration across the endothelium, and increases BBB tightness	miR-98 and let-7g* diminishes levels of pro-inflammatory cytokines by targeting secreted monocyte CCL2 and CCL5	[186]
	2017	miR-155	Up	Mice	miR-155 deficiency improves survival and preserves BBB integrity in experimental cerebral malaria	miR-155 deficiency decreases inflammation and endothelial activation	[187]
	2018	miR-1303	Down	Monkeys, HUVECs	CA16 infection increases the degradation of junctional complexes (claudin-4, claudin-5, VE-cadherin, and ZO-1)	miR-1303 directly targets MMP9, CA16 penetrates the BBB by downregulating miR-1303 and upregulating MMP9	[188]
Diabetes	2017	miR-Let7A	Down	bEnd.3	miR-Let7A overexpression attenuates EC monolayer integrity under high glucose condition	miR-Let7A overexpression prevents cell death and loss of TJ proteins (claudin-5 and ZO-1) and attenuates proinflammatory response and nitrite production	[195]
	2019	miR-200a	Down	Mice, hBMECs	Diabetes increase HDAC3 expression, and HDAC3 inhibition reduces diabetes-induced BBB permeability and rescues junctional protein expression in db/db mice	HDAC3 inhibition is protective against BBB permeability via miR-200a/Keap1/Nrf2 axis in db/db mice	[196]
SAE	2020	miR-181b	Up	Rats, rBMECs, rat brain astrocytes	miR-181b downregulation reduces BBB damage	miR-181b negatively targets S1PR1 and NCALD to worsen BBB impairment	[198]
	2020	miR-370-3p	Up	Mouse and patient samples	CLP-induced SAE increases mouse brain and plasma miR-370-3p levels with BBB permeability deficits	High plasma miR-370-3p was associated with elevated TNF-a and brain apoptosis	[60]
	2021	miR-126	Down	Rats	miR-126 overexpression improves the neurobehavioral score, reduces the brain tissue water content and BBB permeability	miR-126 overexpression increases claudin-5 and occludin, decreases TNF-α, IL-6, IL-1β, and increases IL-10 by inhibiting the NF-kB signaling pathway	[199]
Shear stress	2017	miR-27b	Up	Mice	miR-27a/b inhibition reduces pericyte adhesion, pericyte coverage, and barrier functions, and increases the water content and vessel permeability	Shear stress-regulated miR-27b promotes the interaction of endothelial cells with pericytes, partly by repressing repulsive proteins SEMA6A and SEMA6D	[200]
Development	2017	miR-285	Required for BBB	Drosophila	Loss of miR-285 leads to defective BBB with increased subperineurial glia (SPG) ploidy and disruptive septate junctions	miR-285 directly targets the Yki cofactor Mask (Multiple Ankyrin repeats Single KH domain (Mask)) to suppress Yki activity and down-regulates the expression of its downstream target cyclin E	[201]
Development	2017	miR-132	Required for BBB	Zebrafish larvae, primary rat brain cells	miR-132, secreted from neurons, regulates the brain vascular integrity by affecting adherens junction proteins rather than transcytosis or pericytes	miR-132 regulates VE-cadherin by directly targeting eukaryotic elongation factor 2 kinase (eef2k) to maintain brain vascular integrity	[202]
Aging	2020	miR-195	Down	Mice	miR-195 downregulation increases BBB leakage, miR-195 overexpression improves BBB integrity	miR-195 suppresses thrombospondin-1 (TSP1), which activates selective autophagy to decrease claudin-5 and ZO-1	[203]
Tuberous sclerosis complex	2020	miR-146a and 147b	Not detected	Patient brains, tuber-derived cultures	BBB dysfunction with increased albumin and CD163 is associated with high levels of MMPs and TIMPs (protein expression of MMP2, 3, 9, and 14 and TIMP1, 2, 3, and 4) in cortical tubers	miR-146a and miR-147b can rescue IL-1b-induced dysregulation of MMP3, TIMP2, TIMP3, and TIMP4 in tuber-derived TSC cultures	[204]
RCVS	2021	miR-130a-3p	Up	hCMEC/D3	High levels of circulating miR-130a-3p is associated with BBB disruption in patients with reversible cerebral vasoconstriction syndrome, miR-130-3p overexpression induces EC permeability	miR-130a-3p increases BBB permeability in a dose-dependent manner	[205]
May be related to COVID-19	2021	miR-24	Not detected	hBMECs	miR-24 overexpression reduces VEGFA-induced endothelial permeability	miR-24 directly targets and negatively regulates Neuropilin-1	[208]

**Table 2:** LncRNAs in the regulation of BBB functions in different CNS disorders

CNS disorders	Year	LncRNAs	Levels in disease	Study materials	Main regulatory effects on BBB	Main mechanisms	Refs.
Ischemic stroke	2017	Malat1	Up	BMECs	Malat1 upregulation promotes autophagy in BMECs to promote survival	Malat1/miR-26b/ULK2 regulatory axis promotes autophagy and survival in BMECs	[95]
	2017	Malat1	Up	Mice, mBMECs	Silencing of Malat1 increases apoptotic factor Bim and proinflammatory cytokines MCP-1, IL-6, and E-selectin. Malat1 KO increases brain infarct size, worsens neurological scores, and reduces sensorimotor functions	Malat1 binds to Bim and E-selectin to play anti-apoptotic and anti-inflammatory roles	[96]
	2018	LOC102640519	Up	Mice, mBMECs	Administration of VEGF upregulates LOC102640519 and aggravates BBB permeability	LOC102640519 positively regulates the expression of HOXC13, thus negatively regulates the expression of ZO-1, occludin, and claudin-5	[98]
	2019	MEG3	Up	Rats	MEG3 inhibition ameliorates neurological impairments, reduces infarct area, water content, BBB permeability, neuronal apoptosis and necrosis, and enhances neurogenesis	MEG3 affects neurological injury by regulating Wnt/β-catenin signaling pathway	[99]
	2020	Malat1	Up	Mice, astrocytes	Knockdown of MALAT1 increases cell viability and reduces cell apoptosis in astrocyte cells	MALAT1 acts as competing endogenous RNA (ceRNA) for miR-145 to positively regulate AQP4 expression	[97]
	2021	Snhg8	Down	Mice, BMECs	Snhg8 upregulation increases ZO‐1 and occludin, promotes angiogenesis, inhibits microglial activation after MCAO, and reduces inflammatory factor IL‐1β, IL‐6, and TNF‐α	Snhg8 serves as a ceRNA by sponging miR‐425‐5p to regulate sirtuin1-mediated NF-κB pathway	[100]
	2021	XIST	Down in the early stages	Mice, BMECs	Silencing of lncRNA XIST impairs angiogenesis, reduces the expressions of KLF4 and TJPs (claudin-5 and ZO-1), but increases E-selectin, VCAM-1, ICAM-1, and p-NF-kB	lncRNA XIST upregulates the expression of proangiogenic factor-integrin a5 (Itga5) and anti-inflammation factor KLF4 by targeting miR-92a	[61]
Hemorrhagic stroke	2019	Snhg3	Up	Mice, BMVECs	Snhg3 inhibition improves cell proliferation and migration and reduces cell apoptosis and monolayer permeability in vitro, and improves behavioral scores, BBB integrity, brain water content and cell apoptosis in vivo	Snhg3 regulates cerebral microvascular cell injury through Snhg3/TWEAK/Fn14/STAT3/MMP-2/-9 pathways	[112]
	2021	Blnc1	Up	Mice, BMVECs	Blnc1 overexpression suppresses cell viability and migration but increases permeability, apoptosis, and inflammation in vitro, and its suppression ameliorates nerve injury, brain edema, BBB permeability, and the levels of inflammatory cytokines in vivo	Blnc1 exacerbates nerve injury and inflammatory response through PPAR-γ/SIRT6/FoxO3 pathway	[113]
TBI	2021	KCNQ1OT1	Up	Mice, BV2 microglia	KCNQ1OT1 knockdown relieves neurological deficits, neuron loss, microglial activation and inflammation, and BBB permeability	KCNQ1OT1 inhibition is neuroprotective against TBI in mice by regulating the miR-873-5p/TRAF6/P38/NF-κB axis	[120]
SCI	2021	TUG1	Up	Rats	Knockdown of TUG1 alleviates BSCB leakage and improves hind-limb motor function	TUG1 targets miR-29b-1-5p to regulate inflammatory damage mediated by MTDH/NF-κB/IL-1b pathway	[133]
Glioma/Brain metastasis	2015	TUG1	Up	Patient glioma tissues, GECs	Knockdown of TUG1 increases BTB permeability, and downregulates the expression of the TJ proteins ZO-1, occludin, and claudin-5	TUG1 influences BTB permeability via miR-144/Heat shock transcription factor 2/TJ proteins pathway	[153]
	2016	MALAT1	Up	Patient glioma tissues, GECs	Knockdown of MALAT1 increases BTB permeability of BTB and decreases the expression of ZO-1, occludin, and claudin-5	MALAT1 reciprocally represses miR-140, and that MALAT1 regulates BTB permeability via miR-140/nuclear factor YA/TJ proteins axis	[155]
	2017	Lnc-BM	Up	Mouse	Lnc-BM elevation induces BCBM, while depletion of Lnc-BM inhibits BCBM	LNC-BM increases the cell migration through BBB, and enhances BCBM via Lnc-BM/STAT3/ICMA-1 and CCL2 axis	[62]
	2017	TUG1	Up	Patient glioma tissues, GECs	Knockdown of TUG1 suppresses tumor-induced EC proliferation, migration, and tube formation, and reduces spheroid-based angiogenesis	TUG1 influences tumor angiogenesis via TUG1/miR-299/VEGFA axis	[154]
	2017	XIST	Up	GECs	XIST knockdown increases BTB permeability and inhibits glioma angiogenesis	XIST knockdown increases BTB permeability and inhibits glioma angiogenesis by inhibiting Forkhead Box C1 and ZO-2 expression via increasing miR-137	[157]
	2017	NEAT1	Up	GECs	NEAT1 knockdown increases the BTB permeability, accompanied by downregulated TJ proteins ZO-1, occludin and claudin-5	NEAT1 influences BTB permeability via miR-181d-5p/SOX5/ZO-1, occludin and claudin-5 axis	[158]
	2017	HOTAIR	Up	GECs	Knockdown of HOTAIR increases BTB permeability and downregulates ZO-1, occludin, claudin-5	HOTAIR regulates BTB permeability probably via HOTAIR/miR-148b-3p/USF1 axis	[159]
	2019	linc00174	Up	GECs	Knockdown of linc00174 increases BTB permeability and reduces the expression of TJ-related proteins ZO-1, occludin, and claudin-5	linc00174 regulates BTB permeability via miR-138-5p and miR-150-5p/FOSL2 (FOS Like 2)/ZO-1, occludin, claudin-5 axis	[160]
	2020	GS1-600G8.5	Up	BMECs	lncRNA GS1-600G8.5 highly expressed exosomes increases BBB permeability, and promotes invasion of the breast cancer cells in the BBB model	lncRNA GS1-600G8.5 transferred from exosomes of brain metastatic breast cancer cells might destroy the BBB system by decreasing TJ proteins	[163]
	2020	MIAT	Up	GECs	MIAT promotes the endothelial leakage of BTB	MIAT increases BTB permeability via miR-140-3p/ZAK/NF-κB-p65/TJ associated proteins axis	[164]
	2020	Lnc00462717	Up	GECs	Knockdown of Lnc00462717 significantly increases the BTB permeability	Lnc00462717 reduces the permeability of the BTB through interaction with PTBP1 to inhibit the miR-186-5p/occludin signaling pathway	[161]
	2021	CCRR	Up	Patient tissue, CSF	The expression of lncRNA-CCRR was positively correlated with the up-regulated expression of CX43	lncRNA-CCRR can upregulate the expression of CX43, and promote gap junction formation in brain metastasis of breast cancer	[162]
MS	2021	Gm13568	Up	mice, astrocytes	Inhibiting Gm13568 in astrocytes ameliorates inflammation and demyelination in EAE mice	Knockdown of lncRNA Gm13568 inhibits the Notch1 expression, astrocytosis, and the phosphorylation of STAT3 (p-STAT3), and the production of inflammatory cytokines and chemokines in astrocytes	[174]
AD	2020	LINC00662	Up	BMECs	Knockdown of TRA2A or LINC00662 decreases BBB permeability	TRA2A increases the stability of LINC00662, and LINC00662 decreases ELK4 expression through SMD pathway to downregulate the expression of ZO-1, occludin, and claudin-5	[183]
Bacterial meningitis	2021	NEAT1	Up	Mouse, hCMEC/D3, glioma cells	Downregulation of NEAT1 alleviates BBB damage	NEAT regulates the permeability of BBB via NEAT/miR-135a/HIF1α/ZO-1, occludin, and claudin-5 axis	[189]
	2021	LncRSPH9-4	Up	hBMECs	lncRSPH9-4 overexpression in hBMECs mediates the BBB disruption	Infection induced lncRSPH9-4 upregulates the BBB permeability via miR-17-5p/MMP3/ZO-1, occludin and claudin-5 axis	[190]

**Table 3:** CircRNAs in the regulation of BBB functions in different CNS disorders

CNS disorders	Year	LncRNAs	Levels in disease	Study materials	Main regulatory effects on BBB	Main mechanisms	Refs.
Ischemic stroke	2018	circDLGAP4	Down	Mouse, bEnd.3	Upregulation of circDLGAP4 expression significantly attenuates neurological deficits and decreases infarct areas and BBB damage, and downregulates the EndoMT	circDLGAP4 ameliorates ischemic stroke outcomes by targeting miR-143 to downregulate EndoMT and increase TJ proteins claudin-5, occludin, and ZO-1	[63]
	2020	circCCDC9	Down	Mice	circCCDC9 overexpression protects the BBB barrier with decreased permeability and brain water content and restores NO production and eNOS expression	circCCDC9 overexpression inhibits apoptosis and the expression of Notch1, NICD (Notch intracellular domain), and Hes1 to suppress the Notch pathway	[102]
Subarachnoid hemorrhage	2021	circARF3	Down	Rats	circARF3 overexpression improves BBB integrity and neurological scores, decreases neuronal apoptosis and microglial activation	circARF3 attenuates BBB destruction in SAH by regulating the miR-31-5p-activated MyD88-NF-κB pathway	[114]
TBI	2021	circLphn3	Down	Mice	circLphn3 overexpression attenuates the hemin-induced BBB permeability	circLphn3 binds to miR-185-5p and regulates the expression of tight junction proteins after TBI	[121]
SCI	2021	circRNAAbca1	Up	mice	miR-135b-5p was the most downregulated miRNA after SCI, circRNAAbca1 and KLF4 were predicted to be its target circRNA and mRNA, respectively	circAbca1 plays a neuroinhibitory role by targeting the miR-135b-5P/KLF4 axis	[134]
Glioma	2019	circ‐USP1	Up	GECs	Knockdown of circ‐USP1 disrupts the barrier integrity, increases its permeability, and reduces claudin‐5, occludin, and ZO‐1 expressions	circ‐USP1 regulates barrier permeability via miR‐194‐5p/FLI1/claudin‐5, occludin, and ZO‐1 axis	[165]
	2019	CircRNA_001160	Up	GECs	Polypyrimidine tract binding protein 1 (PTBP1) promotes the function of circRNA_001160, and double silencing of PTBP1 and circRNA_001160 increases BTB permeability	circRNA_001160 regulates BTB permeability via miR-195-5p/ETV1/claudin-5, ZO-1, and occludin axis	[166]
	2019	cDENND4C	Up	GECs	Knockdown of cDENND4C increases BTB permeability via downregulating the expressions of tight junction-related proteins	cDENND4C regulates BTB permeability via miR-577/ZO-1, occludin, and claudin-1 axis	[167]
MS, EndoMT	2018	circular RNA HECW2	Up	hBMECs	Knockdown of circHECW2 expression inhibits the EndoMT, which may contribute to BBB damage	circHECW2 acts as a sponge for miR-30D to regulate the EndoMT, and increases expression of ATG5 (Autophagy Related 5) and the NOTCH pathway	[175]
	2020	circ_HECW2	Up	hBMECs	circ_HECW2 silencing promotes cell proliferation, suppresses cell apoptosis and EndoMT	circ_HECW2 directly binding to miR-30e-5p to regulate NEGR1 and LPS-induced EndoMT	[176]
Meningitis	2020	circ_2858	Up	hBMECs	Overexpression of circ_2858 decreases the level of TJ proteins and increase BBB permeability	circ_2858 regulated BBB permeability by competitively binding miR-93-5p, thereby inducing the upregulation of VEGFA and downregulation TJ proteins such as ZO-1, occludin, and claudin-5	[191]

### Ischemic stroke

Our group is among the first to investigate the regulatory role of microRNA in cerebrovascular endothelial injury and BBB dysfunction after ischemic stroke. We demonstrated that downregulation of ischemia-induced miR-15a expression in the brain can alleviate apoptotic cell death in cerebral microvessels, and reduce BBB disruption and cerebral infarction in mice transient focal cerebral ischemia [64]. Recently, by using an endothelial cell (EC)-selective miR-15a/16-1 conditional knockout mouse model, we show that endothelial-targeted deletion of the miR-15a/16-1 cluster ameliorated BBB leakage and infiltration of peripheral immune cells after experimental ischemic stroke [65], endothelial miR-15a/16-1 cluster is also demonstrated to be a negative regulator for cerebral angiogenesis and long-term neurological recovery following ischemic stroke [66]. Other miRNAs (Fig. 1) that are involved and exhibit regulatory functions on BBB permeability or integrity after ischemic stroke including miR-29b [53], miR-34a [67–69], miR-150 [70], miR-122 [71], miR-210 [72], miR-21 [73], miR-539 [74], miR-155 [75, 76], miR-149-5p [77], miR-1 [78], miR-132 [79], let-7g*[80, 81], miR-98 [80], miR-126-3p/5p [82], miR-98 [83], miR-668 [84], miR-182 [85], miR-503 [54], let-7f [86], miR-30a [87], miR-376b-5p [88], miR-149-5p [89], miR-92b [90], and miR-141-3p [91].

Among these miRNAs, miR-34a, miR-210, miR-155, miR-212/312, miR-15a/16-1, miR-1, miR-182, miR-503, miR-30a, and miR-141-3p exhibit upregulated expression profile under cerebral ischemic or hypoxic conditions, and generally associate with deteriorated neurological neurobehaviors, large infarct volume, and brain edema, and BBB disruption. Downregulation of these enhanced miRNAs through antagomir or miRNA inhibitors significantly alleviated BBB permeability and infiltration of peripheral immune cells, reduced brain infarction and edema, and improved neurological functions after ischemic stroke. For example, miR-34a expression was significantly elevated in the extracted brain microvascular endothelial cells from ischemic mouse brains at the time point of BBB opening following 1-hour MCAO and reperfusion [69], and overexpression of miR-34a significantly enhanced the BBB permeability by disruption of tight junction protein ZO-1 in vitro [67, 68]. Genetic deletion of miR-34a effectively reduced the BBB leakage, alleviated the disruption of tight junction proteins ZO-1, claudin-5, and occludin, and improved neurological recovery following ischemic stroke [69]. Wang et al. demonstrated that miR-30a was significantly increased under ischemic conditions, and inhibition of miR-30a levels with inhibitor decreased the BBB permeability by preventing the degradation of tight junction proteins occludin and claudin-5, and reduction of zinc transporter ZnT4 in both brain endothelial cells and isolated cerebral microvessels of ischemic mice, which yielded ameliorated infarct volume and improved neurological deficits after cerebral ischemia in mice [87].

On the other hand, miR-29b, miR-122, miR-539, miR-149-5p, miR-132, let-7g/7g*, miR-98, miR-126-3p/5p, let-7f, miR-376b-5p, miR-92b exhibited downregulated expression profile under cerebral ischemic conditions. Restoring their expression level through miR mimics or agomirs can significantly downregulate the BBB permeability by enhancing the tight junction protein expression and pericytes coverage, decreasing pro-inflammatory cytokines, matrix metalloproteinases (MMPs), and apoptotic cell death, etc., to reduce the cerebral infarction, brain edema, and improve the overall neurological outcomes following ischemic stroke. For instance, Pan et al. demonstrated decreased expression of miR-126-3p and miR-126-5p in ischemic mouse brains [82]. Lentiviral mediated overexpression in ischemic brains significantly attenuated the decrease of tight junction proteins ZO-1 and occludin, and reduced IgG leakage to the brain tissue three days after stroke [82]. Overexpression of miR-126-3p and -5p also downregulated the expression of pro-inflammatory cytokines interleukin (IL)-1β and tumor necrosis factor (TNF)-α, accompanied by reduced protein level of cell adhesion molecules VCAM-1 and E-selectin three days after stroke [82]. These effects conferred reduced brain infarction, edema volume, and improved behavioral outcomes following ischemic stroke [82].

In addition, one miRNA was found downregulated and another one was upregulated in ischemic stroke, but the protective effects were achieved by strengthening this dysregulation. miR-150 expression was significantly suppressed under hypoxia or ischemic stroke [92], upregulation of miR-150 exhibited worse BBB permeability and decreased claudin-5 expression in both in vitro and in vivo ischemic stroke models [70]. On the contrary, downregulation of miR-150 expression contributed to BBB protection, infarct volume reduction, and neurological deficit amelioration by regulating the angiopoietin receptor Tie-2 [70]. miR-21 was found significantly elevated in the serum of ischemic stroke patients [93, 94], and upregulation of miR-21 expression by mimics significantly reduced ischemic stroke-induced infarct volume, edema, BBB disruption by decreasing the levels of p38, mitogen-activated protein kinase kinase 3 (MAP2k3), inducible nitric oxide synthase (iNOS), and MMP-9 [73].

Recent studies also demonstrated the functional roles of lncRNAs in the regulation of the BBB permeability and neurological recovery in ischemic stroke. Among these differentially expressed lncRNAs in ischemic stroke, lncRNA metastasis-associated lung adenocarcinoma transcript 1 (Malat1) [95–97], LOC102640519 [98], and maternally expressed gene 3 (MEG3) [99] exhibit increased expression, and lncRNA small nucleolar RNA host gene 8 (Snhg8) [100] and X-inactive-specific transcript (XIST) [61] exhibit decreased expression in brain microvascular endothelial cell (BMEC) cultures, astrocytes, or ischemic brain tissues under hypoxia or cerebral ischemia in mice (Fig. 1). Cerebral microvascular endothelial injuries mediate the initial process of BBB disruption in ischemic stroke. We and others show that lncRNA Malat1 is one of the most highly upregulated lncRNA and plays an essential role in the protection against cerebral microvascular endothelial pathophysiology during ischemic stroke [95, 96, 101]. Li et al. showed that upregulation of Malat1 promoted BMEC autophagy as a protective mechanism to enable endothelial survival under ischemic insults. Mechanistic studies revealed that Malat1 serves as a competing endogenous RNA by sponging miR-26b and upregulating the expression of uncoordinated 51-like kinase 2 (ULK2) [95]. Our group demonstrated that Malat1 exerts anti-apoptotic and anti-inflammatory functions in brain microvasculature to ameliorate ischemic cerebral vascular and parenchymal damages through binding to proapoptotic factor Bim and pro-inflammatory molecule E-selectin both in vitro and in vivo. Silencing Malat1 severely aggravated the injury of primary BMEC cultures, and worsened neurological scores, sensorimotor functions, and brain infarct size in the mouse model of ischemic stroke [96]. Astrocytes provide structural and nutritional supports for neurons and are important components of the BBB. Wang et al. showed that Malat1 expression was highly upregulated in astrocytes and animal models of ischemic stroke, however, they observed knockdown of Malat1, instead of overexpression, could protect against ischemia-induced injuries by reducing the cell apoptosis and increasing the cell viability [97]. Further investigation suggested that lncRNA Malat1 could positively regulate the expression of aquaporin-4 (AQP4) by competitively binding miR-145 to mediate the damage of astrocytes during ischemic stroke [97]. LncRNA XIST was found to exhibit decreased serum levels during the early stages of ischemic stroke patients, and silencing lncRNA XIST significantly decreased the endothelial migration and tube formation, and exacerbated cerebral vascular injury by notably reducing the expressions of krüppel-like factor 4 (KLF4) and tight junction proteins claudin-5 and ZO-1, leading to larger infarction and worse neurological functions in transient ischemic stroke mice [61].

Moreover, several circular RNAs have been demonstrated to exert protective function against ischemic stroke-induced BBB disruption in cellular and experimental animal models. Circular RNA DLGAP4 (circDLGAP4) has been found to significantly decrease in the plasma of ischemic stroke patients and in a mouse stroke model, and upregulation of circDLGAP4 expression significantly attenuated neurological deficits and decreased infarct areas and BBB damage, including reduced Evans blue extravasation, ameliorated the downregulation of tight junction proteins claudin-5, occludin, and ZO-1 [63]. CircDLGAP4 also acts as an endogenous miR-143 sponge via targeting miR-143 to downregulate endothelial to mesenchymal transition (EndoMT), an inflamed pathological condition of endothelial cells, to regulate BBB integrity under cerebral ischemic conditions [63]. The expression of circular RNA CCDC9 (circCCDC9) also decreased in ischemic stroke mice, overexpression of circCCDC9 exhibited BBB protection with decreased Evens blue leakage and brain water content, restored NO production and endothelial nitric oxide synthase (eNOS) expression in the ischemic brains. Also, overexpression of circCCDC9 inhibited apoptosis and the Notch pathway by repressing the modulator levels of Notch1, NICD, and Hes1 after cerebral ischemia/reperfusion injury in mice [102].

### Hemorrhagic stroke

Hemorrhagic stroke can lead to severe BBB permeability, neuroinflammation, and cerebral edema. Accumulating investigations have revealed the participation of microRNAs in the regulation of BBB integrity during experimental hemorrhagic stroke models. Among these up or downregulated miRNAs in hemorrhagic stroke, miR-130a [55], miR-21-5p [103], miR-103-3p [104], and miR-24 [105], exhibited upregulated expression profiles under cerebral hemorrhagic conditions (Fig. 1), and inhibition of their expression exert reduced edema, alleviated BBB permeability, improved long-term neurobehavioral functions. For instance, miR-21-5p was found to exhibit elevated serum levels in elderly intracerebral hemorrhage (ICH) or patients, and intracerebroventricular (ICV) administration of miR-21-5p antagomir in aged ICH rats robustly alleviated BBB permeability, inhibited neuronal apoptosis and neuroinflammation, accelerated hematoma absorption, and enhanced neurological and cognitive functions [103]. Dual-specificity phosphatase 8 (DUSP8) was identified as a direct target of miR-21-5p, inhibition of miR-21-5p upregulated the expression of DUSP8, which induced the activation of the p-ERK /heme oxygenase-1 (HO-1) pathways to alleviate hemorrhagic injuries [103]. miR-103-3p also exhibits increased levels in a rat subarachnoid hemorrhage (SAH) model. Repression of miR-103-3p in rats significantly relieved caveolin-1 (Cav-1) loss, reduced BBB permeability, preserved microvascular integrity, and improved long-term neurobehavioral function after SAH [104]. Mechanistic studies demonstrated that miR-103-3p could directly target Cav-1 to decrease the protein expression of ZO-1 and occludin in rats following SAH insults [104].

On the other hand, miR-126-3p [106–108], miR-27a-3p [109], miR-193b-3p [110], and miR-26a-5p [111] exhibited downregulated expression (Fig. 1), restoring or upregulating their levels also yield beneficial effects on BBB integrity and cell survival, neuroinflammatory environment, brain infarction, edema, and neurological outcomes. Several groups investigated the beneficial effects of miR-126-3p against BBB disruption and associated neuronal injuries following ICH. Xi et al. showed that silencing of miR-126-3p led to impaired BMEC barrier integrity, reversed vascular endothelial growth factor (VEGF)- and angiopoietin-1 (Ang-1)-induced Akt activation and apoptosis inhibition [106]. On the other hand, overexpression of miR-126-3p suppressed the upregulation of phosphoinositide-3-kinase regulatory subunit 2 (PIK3R2) and maintained the activation of Akt in the perihematomal area, accompanied by inhibited neutrophil infiltration, microglial activation, and neuronal apoptosis [106]. Fu et al. demonstrated that miR-126-3p showed downregulated expression in serum and hemorrhagic area in ICH rats and downregulated serum expression in patients with ICH [107]. Inhibition of miR-126-3p impaired endothelial barrier permeability by upregulating VCAM-1 expression levels in rat BMECs, while overexpression of miR-126-3p downregulated VCAM-1 expression in the hemorrhagic area in rats following ICH [107]. Wang et al. investigated the efficiency of miR-126-3p-overexpressed bone marrow mesenchymal stem cells (MSCs) in the repair of BBB damage after ICH. They observed that miR-126 facilitated the differentiation of MSCs into vascular endothelial cells in ICH models both *in vitro* and *in vivo* [108]. They also confirmed that miR-126-overexpressed MSC alleviated the cell apoptosis, robustly reduced the expression of protease-activated receptor-1 (PAR-1) and MMP-9, while enhancing the expression of ZO-1 and claudin-5 to improve the neurological outcomes, alleviated brain water content and BBB leakage after ICH [108]. Lai et al. explored the protective effects of miR-193b-3p on early brain injury after SAH. Authors demonstrated that systematic exosomal miR-193b-3p administration effectively suppressed the expression and activity of histone deacetylase 3 (HDAC3) and upregulated the acetylation of NF-κB p65, which reduced the inflammatory cytokine expression in the hemorrhagic mouse brains, and mitigated BBB permeability, brain edema, neurodegeneration, and neurobehavioral impairments after SAH [110].

Two lncRNAs, including lncRNA small nucleolar RNA host gene 3 (Snhg3), and brown fat-enriched lncRNA 1 (Blnc1), were also found to exert BBB regulatory functions during the pathogenesis of ICH. Both LncRNA Snhg3 and Blnc1 exhibited enhanced expression under experimental ICH models (Fig. 1). Downregulation of Snhg3 improved endothelial cell proliferation and migration abilities and attenuated the apoptosis and monolayer permeability in BMVECs under oxygen and glucose deprivation (OGD) with hemin, an in vitro model of ICH [112]. Similarly, downregulation of Snhg3 in vivo improved the integrity of BBB and neurobehavioral scores, while mitigating brain water content and cell apoptosis [112]. Mechanistically, lncRNA Snhg3 can enhance the expression of TWEAK (tumor necrosis factor-like weak inducer of apoptosis) protein and its receptor Fn14 (fibroblast growth factor-inducible 14) to activate the downstream neuroinflammatory pathway STAT3, thus enhancing the secretion of MMP-2 and MMP-9, which contribute to cerebral microvascular dysfunction in ICH rats [112]. Xie et al. observed that inhibition of Blnc1 promoted endothelial cell viability, migration, and endothelial monolayer integrity *in vitro*. Also, suppression of Blnc1 ameliorated BBB permeability and pro-inflammatory cytokines levels, and reduced brain edema in ICH-challenged mice. Blnc1 was further confirmed to be able to positively regulate peroxisome proliferator-activated receptor gamma (PPAR-γ) levels, while Blnc1 knockdown suppressed PPAR-γ/Sirtuin 6 (SIRT6)-mediated forkhead box O-3 (FoxO3) signaling pathway in ICH mice [113].

Circular RNA circARF3 was also revealed to regulate BBB permeability in subarachnoid hemorrhage. Cai et al. observed significant downregulation of circARF3 in plasma and cerebrospinal fluid (CSF) in SAF patients with higher Fisher stages [114]. Overexpressing circARF3 robustly improved BBB integrity and neurological score, and decreased neuronal apoptosis and microglial activation in the ipsilateral basal cortex of SAF rat brains. Mechanistically, the authors demonstrated that circARF3 acted as an endogenous competitive RNA to sponge miR-31-5p, thus deactivating myeloid differentiation factor 88 (MyD88)-NF-κB pathway, to exert protection against SAH-induced BBB disruption [114].

### Traumatic brain injury

Traumatic brain injury (TBI) causes primary and secondary disruptions of microvessels’ structural and physiological integrity and results in compromised BBB integrity, which facilitates the blood-borne factors entering the brain and causes microglial activation, proliferation, and the production of pro-inflammatory factors [115]. Several miRNAs, including miR-21 [40, 116–118] and miR-9-5p [119], exhibited upregulated expression levels in TBI and were associated with regulating BBB integrity after TBI (Fig. 1). Ge et al. demonstrated that upregulation of miR-21 levels in rat brains conferred improved neurological recovery, alleviated BBB permeability, and reduced brain edema and lesion volume following TBI [116–118]. miR-21 can exert BBB protection against TBI injuries via activating the expression of VEGF and Ang-1/Tie-2 to promote the expression of tight junction proteins, such as occludin and claudin-5 to amplify BBB stabilization [116–118]. miR-21 was also able to inhibit the expression of PTEN (phosphatase and tensin homolog deleted on chromosome 10) and activate Akt signaling pathway to inhibit cellular apoptosis [116, 118]. In addition, miR-21 exert anti-inflammatory functions via suppressing the expression of pro-inflammatory cytokines and the NF-κB signaling pathway after TBI [118]. Wu et al. explored the contribution of miR-9-5p on the recovery of neurological function after TBI. Data revealed that upregulation of miR-9-5p significantly alleviated apoptosis, neuroinflammation, and BBB permeability in rats after TBI [119]. Authors also confirmed that miR-9-5p exerted these protective functions through targeting protein patched homolog 1 (Ptch-1) and activating the Hedgehog/AKT/Glycogen synthase kinase-3β (GSK3β) axis to inhibit the NF-κB and MMP-9 signaling pathways [119].

LncRNA KCNQ1 overlapping transcript 1 (KCNQ1OT1) was also involved in the pathogenesis of TBI and played a role in the regulation of BBB permeability after TBI. Liu et al. showed that KCNQ1OT1 was markedly overexpressed in the cerebral tissue of TBI mice, and knockdown of KCNQ1OT1 in the mouse brain exhibited alleviated neurological deficits, neuronal loss, microglial activation, pro-inflammatory cytokines expression (e.g., IL-1β, TNF-α, IL-6, etc.), and augmented anti-inflammatory cytokines (e.g., IL-10, transforming growth factor β (TGFβ), brain-derived neurotrophic factor (BDNF)) accompanied by improved BBB integrity and functions [120]. Authors also confirmed that miR-873-5p was a direct target of KCNQ1OT1, which functioned as a competitive endogenous RNA to sponge miR-873-5p, thus knocking down the levels of KCNQ1OT1 in the brain effectively decreased the levels of tumor necrosis factor receptor-related factor 6 (TRAF6) [120].

Circular RNA Lphn3 (circLphn3) was also demonstrated BBB protection in a cellular and mouse model of TBI. Cheng et al. observed that the expression of circLphn3 was substantially decreased after TBI both *in vivo* and *in vitro* [121]. Overexpression of circLphn3 enhanced the expression of tight junction proteins ZO-1, ZO-2, and occludin, to attenuate the hemin-induced high endothelial permeability in the *in vitro* model of BBB [121]. Authors confirmed that circLphn3 acted as a molecular sponge of miR-185-5p to upregulate the tight junction protein ZO-1 and repair the permeability of BBB after TBI [121].

### Spinal cord injury

The blood-spinal cord barrier (BSCB) functions as the conceptually equivalent to BBB in the spinal cords and provides a similar functional microenvironment as BBB for the cellular constituents of spinal cords; thus, BSCB has been considered as the morphological extension of the BBB [122]. Spinal cord injury (SCI) also results in direct vascular damage and induces prominent disruption of the BSCB [123]. In this context, a number of miRNAs, including miR-27a [124], miR-129-5p [125], miR-199a-5p [126], miR-128-3p [127], miR-9 [128], have been demonstrated to exert protective effects against BSCB damage, but exhibited downregulated levels after the onset of SCI (Fig. 1). For instance, intrathecal injection of miR-129-5p mimics successfully preserved the motor function and prevented the BSCB leakage with decreased Evan blue extravasation and spinal water content [125]. Authors demonstrated that overexpression of miR-129-5p significantly reduced its molecular target high-mobility group box-1 (HMGB1), thus inhibiting Toll-like receptor (TLR)-3, IL-1β, and TNF-α levels in the injured mouse spinal cords [125]. Wang et al. showed that miR-125a-5p overexpression induced the expression of ZO-1, occludin, and VE-cadherin, which lessened the endothelial permeability and cell death in an *in vitro* model of SCI, suggesting miR-125a-5p as anti-apoptotic mediator in spinal cord microvascular endothelial cells after SCI [129].

Several miRNAs exert protective effects on the BSCB structure and function when they are downregulated during SCI. These miRNAs include miR-155 [56, 130], miR-429 [131], and miR-181c-5p [132] (Fig. 1). Awad et al. observed that aortic cross-clamping (ACC)-paralyzed mice exhibit higher miR-155 expression in neurons and endothelial cells than ACC mice that escaped paralysis [56]. Genetic deletion of miR-155 improved BSCB integrity, reduced central cord edema, and reduced total paralysis incidence [56]. Authors elucidated that depletion of miR-155 enhanced the protein levels of major facilitator superfamily domain-containing 2a (Mfsd2) in ECs and motoneurons to attenuate EC permeability and grey matter damage [56]. In addition, Ge et al. also confirmed that miR-155 might aggravate BSCB disruption following experimental SCI [130]. Authors showed that exosomal miR-155 from M1-polarized macrophages promotes EndoMT, a pathological condition of ECs, and impairs mitochondrial function via activating NF-κB signaling pathway after traumatic SCI by directly targeting downstream suppressor of cytokine signaling 6 (SOCS6) and inhibiting the expression of tight junction proteins ZO-1, occludin, claudin-1, -2 and -5 in vascular endothelial cells [130]. Sun et al. observed that inhibition of miR-429 expression by antagomir resulted in significantly increased levels of tight junction proteins ZO-1, occludin, and claudin-5 and reduced BSCB permeability in an extracorporeal BSCB model [129]. Authors then elucidated that miR-429 can negatively regulate KLF6 to mediated tight junction protein expression and BSCB integrity [129].

Regarding long non-coding RNA in the regulation of BSCB permeability following experimental SCI, the functional role of lncRNA taurine upregulated gene 1 (TUG1) was investigated. Jia et al. discovered that knockdown of TUG1 alleviated blood-spinal cord barrier leakage and improved hind-limb motor function by upregulating miR-29b-1-5p and suppressing metadherin (MTDH)/NF-κB pathway-mediated inflammatory cytokines expression after spinal cord ischemia-reperfusion [133].

Circular RNA has also been studied in the involvement of the pathophysiology of traumatic SCI. By using the RNA-seq technique, Wang et al. systematically studied the expression profile of circRNA in the lesion epicenter of spinal tissues after traumatic SCI, with a focus on circAbca1 [134]. Authors demonstrated that miR-135b-5p was the most significantly downregulated microRNA, and circAbca1 exhibited significant upregulation after traumatic SCI; authors then confirmed that circAbca1 plays a neuroinhibitory role by the miR-135b-5p/KLF4 axis [134], which may relate to the severe damage to the blood-spinal cord barrier observed after traumatic SCI.

### Glioma, glioblastoma, and CNS metastasis

CNS tumors have grown substantially during the past two decades. Glioma constitutes the most diagnosed brain tumor type among children ages 0 to 14, glioblastoma constitutes the most common type of primary malignant brain tumor and other CNS tumor diagnosed in adults 40 years or older in the United States [135]. Glioblastoma is the most aggressive type of tumor of glioma. CNS metastasis is another type of CNS tumor, which refers to the spread of cancer cells from the primary site of the body to the CNS, and the incidence of CNS metastasis is rapidly increasing in the past decade [136]. The integrity of normal BBB was compromised during the progress of CNS tumors and resulting in a vasculature known as the blood-tumor barrier (BTB) [137]. Although BTB has been characterized as a disrupted BBB, it preserves the critical characteristics of the BBB, which still restrain the easy delivery of therapeutic agents to the tumor tissue [137], thus increasing the permeability of BTB has become a therapeutic strategy for CNS tumors. Emerging evidence has shown the extensively regulatory abilities of non-coding RNAs (miRNAs, lncRNAs, and circular RNAs, etc.) on the BBB/BTB integrity and permeability in experimental models of glioma, glioblastoma, and CNS metastasis (Fig. 1). Small non-coding RNAs that involved in BBB/BTB regulation including miR-181a [138], miR-34c [139], miR-18a [140, 141], miR-34a [142], miR-200b [143], miR-330-3p [144], piR-DQ590027/miR-17HG [145], miR-429 [146], piRNA-DQ593109/miR-330-5p [147], miR-132-3p [148], etc. for glioma and glioblastoma; and miR-105 [58], miR-181c [149], miR-509 [150], miR-101-3p [151], and miR-211 [152], etc. for brain metastasis. For example, it was described that miR-429 exhibited lower expression in glioma endothelial cells (GECs), an in vitro BTB model, and overexpression of miR-429 in GECs significantly decreased the expression of tight junction proteins ZO-1, occludin, and claudin-5, as ZO-1 and occludin were direct targets of miR-429 [146]. In addition, miR-429 can also downregulate the tight junction-associated proteins by targeting p70S6K to increase the BTB permeability in glioma models [146]. It was also reported that the expression of miR-132-3p was greatly upregulated in GECs, and miR-132-3p contributed to the increased permeability of BTB and caveolae-mediated transcellular transport by targeting its downstream molecule PTEN, and positive regulation of phosphorylated protein kinase B (p-PKB), p-Src, and p-Cav-1 (Tyr14 phosphorylation of caveolin-1) [148]. Moreover, in breast cancer brain metastasis, Pan et al. demonstrated that high levels of miR-211 drove early and specific brain colonization of tumor cells to enhance their stemness properties and trans-BBB adherence and migration to promote brain metastasis by downregulating the SOX11 (SRY-Box Transcription Factor 11)/NGN2 (neurogenin 2)-dependent axis *in vivo* [152].

Besides small non-coding RNAs, numerous lncRNAs exhibited upregulated expression in brain tumors, and their inhibition generally yields decreased BTB integrity and increased permeability through different pathways. These lncRNAs includes lncRNA TUG1 [153, 154], Malat1 [155, 156], Lnc-BM [62], XIST [157], NEAT1 (nuclear paraspeckle assembly transcript 1) [158], HOTAIR (homeobox transcript antisense intergenic RNA) [159], linc00174 (long intergenic non-protein coding RNA 174) [160], Lnc00462717 [161], CCRR (cardiac conduction regulatory RNA) [162]. These lncRNAs normally act as competing endogenous RNAs to bind their downstream microRNAs, then regulate the BTB permeability. For instance, Zhang et al. observed that Lnc00462717 was upregulated in GECs, and that knockdown of Lnc00462717 reduced its interaction with PTBP1 (Polypyrimidine Tract Binding Protein 1), thus significantly increasing levels of miR-186-5p to subsequently downregulate the protein expression of occludin to increase the BTB permeability [161]. LncRNA GS1-600G8.5 [163] and MIAT [164] were also highly expressed in brain metastatic cells and GECs, however, instead of suppression, overexpression can facilitate endothelial barrier permeability and promote the invasion of cancer cells across the BBB/BTB. For example, MIAT was shown to function as a miR-140-3p sponge to upregulate the expression of ZAK (ZO-1-associated kinase) and the phosphorylation of NF-κB-p65, to inhibit the expression of tight junction proteins ZO-1, occludin, and claudin-5 to increase permeability in an *in vitro* model of BTB [164].

Furthermore, it was observed that circular RNA USP1 (circ‐USP1) [165], circRNA_001160 [166], and circular RNA DENND4C (cDENND4C) [167] were also deeply involved and markedly upregulated in GECs. Knockdown of the cellular expression of circ‐USP1, circRNA_001160, cDENND4C disrupted the barrier integrity, increased barrier permeability accompanied by reduced tight junction-related proteins claudin-5, occludin, and ZO-1 by targeting miR-194-5p/FLI1 (Friend leukemia virus integration 1), miR-195-5p/ETV1 (Ets variant gene 1), and miR-577, respectively [165–167].

### Multiple sclerosis

Multiple sclerosis (MS) is a chronic, autoimmune disease that affects the normal function of the CNS. A number of studies have investigated the involvement and functional significance of various non-coding RNAs in the regulation of barrier function in MS. Several miRNAs exhibited downregulated expression from MS patients’ brain/spinal cord tissue or blood samples, as well as *in vitro* BBB models treated with pro-inflammatory molecules (Fig. 1). These downregulated miRNAs include miR-125-5p [168], miR-320a [169], and miR-126 & miR-126* [170]. Upregulating their expression levels can generally improve brain endothelial cell barrier function by decreasing the expression of cell adhesion molecules, such as ICAM-1, VCAM-1, and E-selectin, or targeting intracellular MMPs expressions. For example, Cerutti et al. observed that reduction of endothelial miR-126 and miR-126* resulted in enhanced expression of E-selectin and VCAM-1, respectively, to enhance the firm adhesion of leukocytes and primary MS patient-derived peripheral blood mononuclear cell (PBMC) to brain endothelial cells [170]. In contrast, overexpression of miR-126 reduced the expression of VCAM1 and MCP1 (monocyte chemoattractant protein 1) expression in brain microvascular endothelial cells [170].

miR-155 exhibited upregulated cerebral expression in MS patients or experimental models [171–173]. Lopez-Ramirez et al. showed that loss of miR-155 reduced BBB extravasation of both experimental autoimmune encephalomyelitis (EAE) and in an acute systemic inflammation model induced by lipopolysaccharide [172]. The mechanistic investigation demonstrated that miR-155 modulated brain endothelial barrier function by targeting both cell-cell complex molecules, such as annexin-2 and claudin-1, and cell-to-extracellular matrix interactions, such as dedicator of cytokinesis 1 (DOCK-1) and syntenin-1 (SDCBP), to increase the barrier permeability [172]. It was also reported that miR-155 overexpression boosted the levels of VCAM-1 and ICAM-1, which facilitated the firm adhesion of monocytic and T cells to both unstimulated and pro-inflammatory cytokines-stimulated human brain endothelium, thus enhancing the leukocytes extravasation of the inflamed BBB [173].

As astrocytes actively participate in the formation and integrity of the BBB, the function of astrocytic lncRNA Gm13568 in the regulation of MS pathophysiology has been investigated in experimental EAE mice and primary astrocyte culture. Liu et al. observed that inhibiting Gm13568 levels in astrocytes significantly ameliorated inflammation and demyelination in EAE mice, which delayed the progress of experimental EAE [174]. Knockdown of the endogenous Gm13568 in IL-9 treated primary astrocyte culture remarkably suppressed astrocytosis and the phosphorylation of signal transducer and activator of transcription 3 (p-STAT3) as well as the production of inflammatory cytokines and chemokines (IL-6, TNF-α, interferon-inducible protein-10) through inhibiting the Notch1 pathway [174].

Moreover, circular RNA HECW2 (circ_HECW2) [175, 176] was also involved in the pathogenesis of MS. Elevated expression of circ_HECW2 leads to EndoMT, which plays a critical role in the dysfunction of BBB and contributes to BBB leakage, in both *in vitro* and *in vivo* MS experimental models [175, 176]. Yang et al. showed that circ_HECW2 functioned as a miR-30D sponge to increase the expression of ATG5 (autophagy-related 5) and activate the NOTCH pathway, then positively regulate LPS-induced EndoMT [175]. Dong et al. further revealed that circ_HECW2 also interacted with miR-30e-5p to regulate the levels of neuronal growth regulator 1, which repressed endothelial cell proliferation and exacerbated apoptosis and LPS-induced EndoMT [176].

### Dementia

BBB breakdown and pericyte degeneration have been found in vascular cognitive impairment and dementia (VCID) and Alzheimer's disease (AD). In this context, non-coding RNAs have emerged as critical BBB regulators during the pathological process of these CNS disorders. In experimental AD models, it has been reported that miR-107 [177], miR-181a [59], miR-124 [178] showed downregulated expression, while miR-96 [179] and miR-424-5p [180] showed up-regulated expression in AD environments (Fig. 1). In experimental VCID models, it has been shown that miR-126 was downregulated in multiple microinfarction (MMI) model-induced vascular dementia [181], and miR-501-3p was upregulated in bilateral common carotid artery stenosis (BCAS)-induced VCID [182] (Fig. 1). These dysregulated microRNAs are typically associated with decreased endothelial cell viability, impaired BBB integrity, increased BBB permeability, and/or declined microvascular density and angiogenesis. On the other hand, overexpression of miR-107 [177], miR-181a [59], miR-124 [178], or inhibition of miR-96 [179] and miR-424-5p [180] in AD significantly abrogated beta-amyloid-induced cerebrovascular injury and BBB disruption through upregulating junctional protein expression or ameliorating pericyte apoptosis. For example, Zhang et al. reported that miR-96 could target erythroblast transformation-specific (ETS) transcription factor ERG (ETS-related gene) to inhibit ERG protein expression, which can bind to ZO-1 promoter region to downregulate the ZO-1 transcription in BMECs; thus, inhibition of miR-96 prevented ZO-1 downregulation induced by GM-CSF (granulocyte-macrophage colony-stimulating factor) in AD environments [179]. Li et al. observed that C1ql3, one of the C1q subunits, was a potential target of miR-124, and overexpression of miR-124 dramatically elevated the expression of ZO-1 and robustly rescued breakdown of the BBB, promoted angiogenesis and reduced Aβ deposition, and finally alleviated learning and memory deficit in APP/PS1 mice [178]. In an MMI-induced vascular dementia model, Yu et al. discovered that miR-126 was downregulated and negatively regulated MMP-9 and TLR4 inflammatory factor expression in endothelial cells, which are related to BBB disruption and neuroinflammation [181]. EC-targeted deletion of miR-126 exhibited significant water channel and glymphatic impairment [181], suggesting upregulation of miR-126 levels may provide beneficial therapeutic effects against vascular cognitive impairments.

Long non-coding RNA LINC00662 was also able to regulate the BBB permeability in Alzheimer's microenvironment. It was reported that LINC00662 was upregulated in beta-amyloid-incubated microvascular endothelial cells, and knockdown of LINC00662 decreased BBB permeability in AD microenvironment [183]. LINC00662 downregulated the expression of ETS-domain protein 4 (ELK4), which can bind to the promotors of ZO-1, occludin, and claudin-5 to promote their protein expression [183]. Thus, inhibition of LINC006622 resulted in enhanced expression of ELK4, increased the levels of tight junction proteins, and improved BBB integrity in the AD microenvironment [183].

### Bacterial and viral infections

Cerebral infections can be caused by various bacteria, viruses, fungi, parasites, or other inflammatory factors, leading to severe brain inflammation and injuries. The integrity and normal functions of BBB are largely compromised by the infectious microorganisms, resulting in increased BBB permeability and exacerbated brain injuries [184]. In this context, emerging evidence has demonstrated the regulatory function of non-coding RNAs on BBB integrity and permeability during cerebral infections. For instance, Mishra et al. showed that HIV Tat C protein significantly impaired the BBB permeability and decreased the expression of VE-cadherin and tight junction proteins (TJPs) (claudin-5, ZO-1, occludin) [185]. Further investigation observed that HIV Tat C protein increased the expression of miR-101, which led to suppression of VE-cadherin in human BMECs [185]. Rom et al. observed the downregulation of miR-98 and let-7g* in experimental models of aseptic meningitis [186]. Overexpression of let-7 and miR-98 suppressed the secretion of CCL2 and CCL5, reducing the leukocyte adhesion and migration across the endothelium, diminishing the pro-inflammatory cytokines, and improving the BBB integrity [186]. In an experimental mouse model of cerebral malaria, plasmodium berghei ANKA (PbA) infection increased circulating exosomal miR-155 levels [187]. Genetic deletion of miR-155 ameliorated endothelial activation, preserved BBB integrity, and improved survival rate in response to infection in experimental cerebral malaria (ECM) models [187]. miR-155 antagomir administration also reduced *ex vivo* vascular leakage in human cerebral microvessels exposed to sera collected from children with cerebral malaria in Ugandan [187]. In an in *vitro* BBB model with coxsackievirus A16 (CA16) infection and an *in vivo* CA16 infant rhesus monkey infection model, Song and colleagues suggested that CA16 infection downregulated miR-1303 levels and upregulated MMP9 expression, which promoted the degradation of junctional proteins, including claudin4, claudin5, VE-cadherin, and ZO-1, and ultimately causing neuroinflammation and injury to the CNS [188].

Two long non-coding RNAs are involved in the regulation of bacterial meningitis-induced BBB damages. Wang et al. showed that NEAT1 levels were upregulated in glioma-exposed endothelial cells and miR-135a was a direct target of NEAT1 [189]. Downregulation of NEAT1 effectively maintained BBB integrity and decreased BBB permeability in bacterial meningitis experimental models through upregulating miR-135a and downregulating HIF1α to increase the expression of ZO-1, occludin, and claudin-5 [189]. On the other hand, overexpression of NEAT1 increased BBB permeability in both *in vitro* and *in vivo* bacterial meningitis models [189]. In an *in vitro* bacterial meningitis model, Xu et al. demonstrated that long non-coding RNA LncRSPH9-4 was significantly elevated and cytoplasmically distributed in meningitic E. coli-infected hBMECs. LncRSPH9-4 was able to regulate the BBB permeability by competitively sponging miR-17-5p, thereby increasing MMP3 expression, which can target the tight junction proteins ZO-1, occludin, and claudin-5 in meningitic E. coli-infected hBMECs [190].

Yang and colleagues also observed circular RNA circ_2858 mediated the BBB disruption in bacterial meningitis. Data revealed that circ_2858 was significantly upregulated by meningitic E. coli infection in human BMECs, and circ_2858 competitively bound miR-93-5p to elevate VEGFA levels [191]. The enhanced VEGFA expression led to downregulation and altered distribution of tight junction proteins such as ZO-1, occludin, and claudin-5, which eventually increased BBB permeability in bacterial meningitis [191].

### Diabetes

Hyperglycemia and diabetes have been known to induce cerebrovascular stress and trigger BBB impairment and permeability, leading to severe cerebrovascular disorders, such as stroke and dementia [192–194]. It has been reported that several microRNAs participated in BBB regulation under diabetic conditions. Song et al. described that high glucose condition downregulated miR-Let7A expression in brain endothelial cells in vitro. Overexpression of miR-Let7A markedly attenuated endothelial cell death and the loss of tight junction proteins (claudin-5 and ZO-1), diminished the levels of pro-inflammatory factors (TNF-α and iNOS), and nitrite production in the brain endothelial cells under high glucose condition [195]. Zhao et al. demonstrated HDAC3 inhibition reduced diabetes-induced BBB permeability and rescued junction protein expression in db/db diabetic mice, and HDAC3 inhibition-mediated protective effects against BBB permeability was at least partly mediated by miR-200a [196]. Data revealed that HDAC3 inhibition significantly increased the miR-200a levels, which targeted and downregulated Keap1 (Kelch-like ECH-associated protein 1), the negative regulator of Nrf2 (nuclear factor-erythroid factor 2-related factor 2), thereby contributing to Nrf2 activation and ultimately the protection against endothelial monolayer permeability under diabetic conditions [196].

### Sepsis-associated encephalopathy

Blood–brain barrier disruption induced by sepsis plays a critical role in the pathophysiology of sepsis-associated encephalopathy (SAE), which increases the influx and efflux of various circulating immune cells, detrimental pathogens, and harmful molecules between the circulation and the brain [197]. Recently, emerging studies have described the functional significance of microRNAs in regulating BBB permeability in SAE. For example, in a septic rat model, Chen et al. demonstrated that miR-181b was elevated and negatively targeted sphingosine-1-phosphate receptor 1 (S1PR1) and neurocalcin delta (NCALD) [198]. Inhibition of miR-181b levels reduced damage and permeability to the BBB via increasing the expression of S1PR1 and NCALD in septic rats [198]. Visitchanakun et al. showed increased expression of miR-370-3p in plasma and brain tissue was associated with SAE outcomes [60]. Plasma miR-370-3p also specifically increased and highly sensitive for early detection (6h) of cecal ligation and puncture (CLP)-induced SAE with BBB permeability, elevated TNF-a, and brain apoptosis [60]. Additionally, Nong et al. revealed downregulated expression of miR-126 in septic rat brain tissues, and overexpression of miR-126 significantly reduced the brain tissue water content and BBB permeability in SAE rats, and significantly increased the expression of claudin-5 and occludin. Overexpression of miR-126 also decreased the serum levels of pro-inflammatory factors TNF-α, IL-6, and IL-1β and increased the expression of anti-inflammatory IL-10 [199].

### Other CNS disorders

Recent investigations also suggested that microRNAs are involved in regulating blood–brain barrier integrity in other pathophysiological events, including shear stress, development, aging, tuberous sclerosis complex, reversible cerebral vasoconstriction syndrome (RCVS), and others. For instance, shear stress-upregulated miR-27b in endothelial cells increased pericyte adhesion and pericyte recruitment of endothelial tubes to preserve the BBB integrity [200]. miR-285 and miR-132 were demonstrated to be essential to BBB development and to maintain brain vascular integrity [201]. miR-285 directly targets the Yki cofactor Mask (Multiple Ankyrin repeats Single KH domain) to suppress Yki activity and downregulate the expression of cyclin E to regulate cell cycle and keep proper cell size to maintain a functional BBB in Drosophila [201]. miR-132 regulated the brain vascular integrity by affecting adherens junction protein VE-cadherin rather than transcytosis or pericytes in larval zebrafish [202]. In aging, cerebral miR-195 levels decreased with age, and led to increased expression of thrombospondin-1 (TSP1), which can activate selective autophagy of tight junction proteins by increasing the formation of claudin-5-p62 and ZO-1-p62 complexes, resulting in TJ protein degradation and BBB permeability [203]. In tuberous sclerosis complex (TSC), high protein expression of MMPs (MMP2, 3, 9, and 14) and TIMPs (endogenous tissue inhibitors 1, 2, 3, and 4) in TSC tubers was associated with BBB dysfunction, while these dysregulated proteins can be partly rescued by miR-146a and miR-147b in tuber-derived TSC cultures [204]. In reversible cerebral vasoconstriction syndrome (RCVS), high expression of circulating miR-130a-3p was associated with BBB disruption in patients, and overexpression of miR-130a-3p also led to increased BBB permeability in vitro [205]. Neuropilin-1 (Npn-1) has been suggested to play a critical role in regulating endothelial barrier dysfunction in response to VEGF [206] or interferon-γ [207], and Mone et al. demonstrated that Npn-1 is a direct target of miR-24, which could negatively regulate Npn-1 mediated endothelial permeability [208].

## Non-coding RNAs as therapeutic targets of pharmacotherapy in regulating BBB/BSCB functions in CNS disorders

Some pharmaceutical drugs preserve the ability to regulate BBB or BSCB functions by modulating non-coding RNAs, thus making these ncRNAs therapeutic targets in regulating BBB or BSCB integrity and permeability in CNS disorders. As shown in Table 4, we summarized the reported pharmacologic agents that exert BBB regulatory functions in different experimental models of CNS disorders, including ischemic stroke, intracerebral hemorrhage, TBI, SCI, AD, and others. Polydatin (PD), a natural product, has been described to enhance lncRNA MALAT1 gene expression, reduce cell toxicity, apoptosis, and inflammatory factor expression in rat brain microvascular endothelial cells [209]. Also, PD administration in rats elevated MALAT1 expressions, reduced cerebral infarct volume and brain inflammation, protected cerebrovascular endothelial cells and BBB integrity after cerebral ischemia [209]. Similarly, Alisol A 24-acetate (AA), a natural compound, has been observed to downregulate the expression of miR-92a-3p, increase ZO-1, claudin-5, and occludin expression in the OGD-insulted BMECs, which provided evidence for AA application in aging-associated BBB protection [210]. Methamphetamine abuse led to upregulation of miR-143 in isolated brain microvessels and tissues, which was also accompanied by BBB leakage [211]. Silencing miR-143 ameliorated methamphetamine-triggered permeability of endothelial cells monolayer and the BBB both *in vitro* and *in vivo* through targeting PUMA (p53 upregulated modulator of apoptosis), NF-κB, and p53 transcription factor pathway [211]. Salvianolic acid A can improve the recovery of neurological function after SCI, which could be correlated with the repair of BSCB integrity by the miR-101/Cul3 (Cullin 3)/Nrf2/HO-1/ZO-1 and occludin signaling pathway [212]. Hydrogen gas (H_2_) can robustly improve neurological outcomes after TBI by mitigating neurological dysfunction, alleviating brain edema, and decreasing lesion volume and BBB permeability by significantly increasing the expression of miR-21 [213]. Memantine (MEM), an *N*-methyl-d-aspartate (NMDA) receptor antagonist, has been demonstrated to alleviate the drastic increase of circular RNA LINC00094 in beta-amyloid-incubated microvascular endothelial cells in an *in vitro* BBB model. Suppression of LINC00094 levels significantly mitigated BBB permeability and upregulated the expression of ZO-1, occludin, and claudin-5 through the miR-224-5p (miR-497-5p)/Endophilin-1 axis [214].

**Table 4:** Non-coding RNAs mediate BBB regulatory effects of pharmaceutical drugs in CNS disorders

Year	pharmaceutical drugs	Non-coding RNAs	Levels change by agents	CNS Diseases	Study materials	Main regulatory effects on BBB	Main mechanisms	Refs.
2016	Methamphetamine	miR-143	Increased	Methamphetamine abuse	mice, hBMECs	Methamphetamine administration causes BBB damage. Silencing miR-143 ameliorates the increased BBB permeability induced by methamphetamine	Methamphetamine induces expression of miR-143 via sigma-1 receptor/MAPK (mitogen-activated protein kinase)/STAT3 pathway, and miR-143 regulates the EC permeability of endothelial cells via PUMA/NF-κB and p53/TJ proteins axis	[211]
2016	Salvianolic acid A	miR-101	Increased	SCI	rats, rBMECs	Sal A improves the recovery of neurological function after SCI	Sal A repairs BSCB integrity by the miR-101/Cul3/Nrf2/HO-1/ZO-1, occludin, and p-caveolin-1 signaling pathway	[212]
2018	Hydrogen gas (H_2_)	miR-21	Increased	TBI	rats	H_2_ treatment improves neurological dysfunction, alleviates brain edema, decreases lesion volume and BBB permeability	H_2_ treatment decreases the levels of oxidative products and increases the activities of endogenous antioxidant enzymes by upregulating miR-21 expression	[213]
2019	Memantine (MEM)	LINC00094	Decreased	AD	hCMEC/D3	MEM been used widely for AD therapy, and silencing LINC00094 enhances the effect of MEM on decreasing BBB permeability in AD microenvironment	Reduction of LINC00094 inhibits endophilin‐1 expression by upregulating miR‐224‐4p/miR‐497‐5p and promotes the expression of ZO‐1, occludin, and claudin‐5 in AD conditions	[214]
2019	Monomethyl fumarate (MF)	miR-139	Increased	Intracerebral hemorrhage	rats, SH-SY5Y	MF pretreatment markedly alleviates BBB disruption and brain edema	MF protects ICH in rats by inhibiting oxidative stress (increased Nrf2) and inflammatory response (decreased NF-κB) through activating the microRNA-139/Nrf2 axis	[215]
2019	Polydatin (PD)	Malat1	Increased	Ischemic stroke	rats, rBMECs	PD reduces cell toxicity and apoptosis, reduces inflammatory factors, and enhances the expression of BBB markers after OGD. PD reduces cerebral infarct volume and brain inflammation, protects cerebrovascular endothelial cells and BBB integrity after cerebral ischemia	PD activates the MALAT1/CREB (cAMP-response element binding protein)/PGC-1α (Peroxisome proliferator-activated receptor-gamma coactivator-1alpha)/PPARγ signaling pathway to protect endothelial cells against ischemia	[209]
2021	Alisol A 24-acetate (AA)	miR-92a-3p	Decreased	Ischemic stroke	BMECs	AA enhances cell viability and increases ZO-1, claudin-5, and occludin expression in OGD-insulted BMECs	AA protects against BMECs damage and TJ proteins loss through the inhibition of miR-92a-3p expression	[210]

## Challenges, perspectives, and future goals

**Fig. 2:**
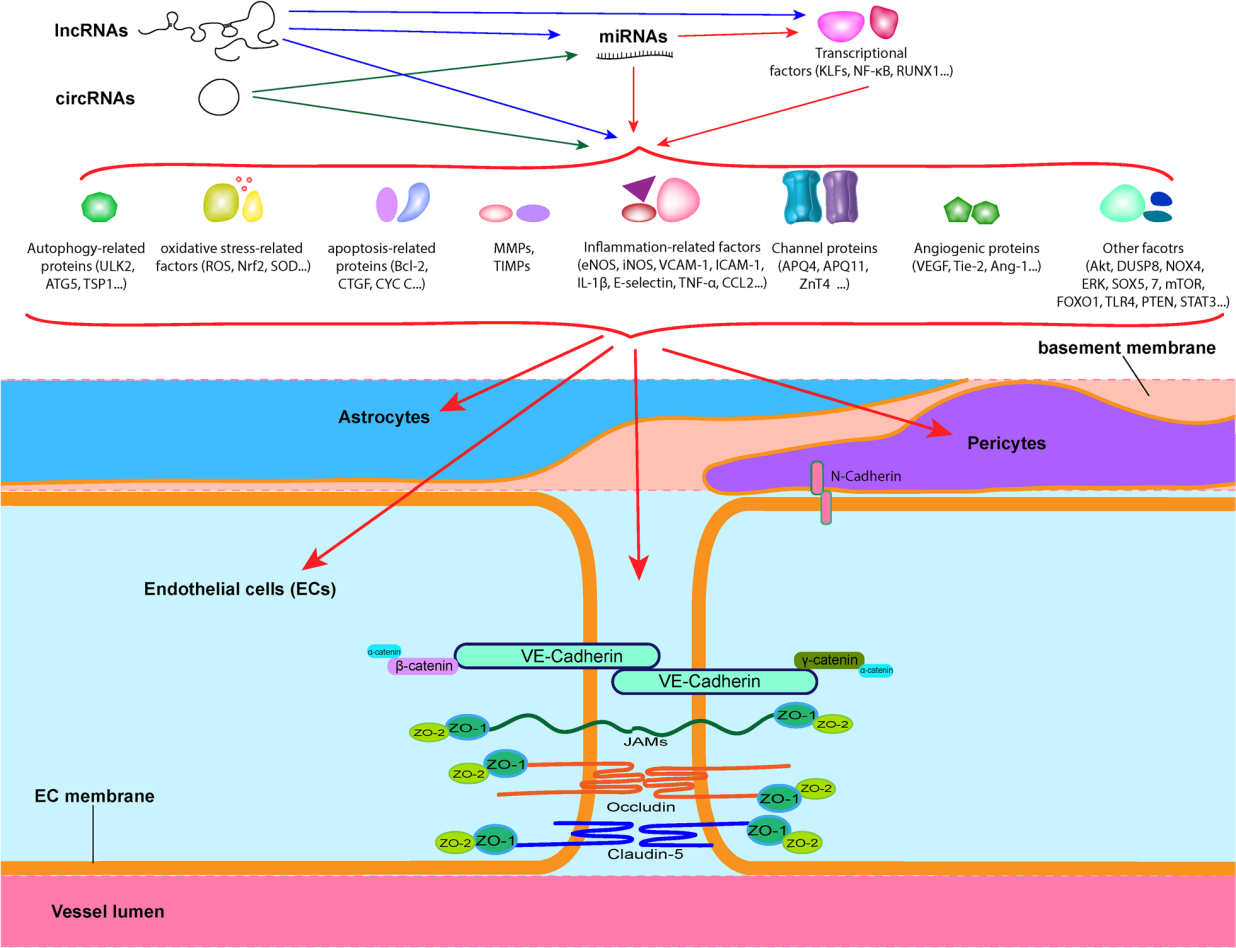
Summarized mechanisms of BBB regulatory miRNAs, lncRNAs, and circRNAs in CNS disorders. MiRNAs, lncRNAs, and circRNAs modulate the integrity of BBB through different mechanisms, including direct or indirect modulation of tight junction proteins, adherens junction proteins, MMPs, water channel-related proteins, angiogenesis-related proteins, inflammation-related factors, apoptosis-related molecules, oxidative stress-related factors, autophagy-related proteins, and others, in endothelial cells, pericytes, or astrocytes of the CNS. LncRNAs and circRNAs primarily act as microRNA “sponges” or competing endogenous RNAs to regulate BBB damage and recovery in CNS diseases

BBB dysfunction is a common pathological feature upon the onset of various CNS diseases, and the disrupted BBB further exacerbates the initial CNS damage. BBB damage has become a vital factor in determining the progression and outcomes of CNS disorders. Currently, there is no available clinical pharmacotherapy in the treatment of the BBB dysfunctions directly [216]. The involvement and regulatory functions of non-coding RNAs on BBB dysfunction in CNS disorders have been rapidly and vastly investigated during the past decade. Considerable evidence has demonstrated the effectiveness and ability of miRNAs, lncRNAs, and circRNAs in the protection of the BBB/BSCB in stroke, TBI, SCI, MS, dementia, brain infections, diabetes, SAE, and others, and in the enhancement of BTB permeability to facilitate the anti-cancer drug delivery in glioma and brain metastasis. As shown in Fig. 2, miRNAs, lncRNAs, and circRNAs modulate the integrity of BBB through a number of mechanisms, including directly or indirectly modulate TJ proteins (mostly claudin-5, occludin, and ZO-1), AJ proteins, MMPs, water channel-related proteins, angiogenesis-related proteins, inflammation-related factors, apoptosis-related molecules, oxidative stress-related factors, autophagy-related proteins, and others, in endothelial cells, pericytes, or astrocytes of the CNS. LncRNAs and circRNAs primarily act as microRNA “sponges” or competing endogenous RNAs to participate in BBB regulation in CNS diseases. There are also increasingly discovered agents in regulating BBB/BSCB functions in CNS disorders through the functions of ncRNAs. Thus, these studies have shed light on the discovery of novel pharmaceutical drugs for the treatment of BBB impairments in numerous CNS diseases. However, nowadays, the most commonly used drugs to treat BBB dysfunctions are glucocorticoids that are able to improve the tightness and contribute to BBB stabilization [216–218], and numerous difficulties and challenges exist on the translation of non-coding RNA-based therapeutics from bench to bedside.

One challenge might be the side-effects of the usage of one non-coding RNA in the disease. As one ncRNA can target numerous different nucleotides or molecules, such as microRNA [219], some ncRNAs play different or even opposite roles on BBB integrity among different CNS diseases, and the most used methods to experimentally modulate the expression of ncRNAs are mimics or inhibitors with systematic administration or cell cultures, strategies to increase the specificity of ncRNAs to their targets, cell types, location are critical for the efficiency and efficacy of ncRNA-based therapeutics. Also, the relationship between the expression levels of dysregulated ncRNAs and the severity of BBB/BSCB/BTB leakage in CNS disease needs to be better investigated.

Another challenge is searching for the origin of the dysregulated ncRNAs after CNS diseases. Most investigations utilize the whole blood, whole or part of CNS tissues to analyze the levels of dysregulated ncRNA, but lack of search for the actual origin (e.g., cell type, tissue type), and how did the dysregulated ncRNA transport to the affected region of the CNS. Interestingly, some studies have focused on the functional roles of cell-specific ncRNAs on BBB or cerebrovascular systems after CNS injuries [65, 66, 181]. Other studies have investigated the transportation of ncRNA from one type of neuronal cells to BBB components and the regulatory mechanisms for the BBB integrity in CNS diseases [130, 149, 163, 202].

As to investigating the roles of ncRNAs in the regulation of BBB, most studies focused on TJ proteins, especially claudin-5, occludin, and ZO-1. Other TJ proteins, AJ proteins, JAMs, and cytoplasmic accessory proteins are critical elements as well to build an intact and functional BBB, thus warranting more research efforts in the regulation of BBB by ncRNAs in CNS diseases. Moreover, pericytes and astrocytes are crucial for maintaining BBB integrity and function, but only a few studies have been involved regarding ncRNA-regulated roles in CNS diseases. For example, Wan et al. show the functions of miR-149-5p in pericytes on BBB integrity after ischemic stroke [77], Wu et al. demonstrate the protective role of miR-181a in pericytes on BBB breakdown in AD [59], and Wang et al. validate the functional role of lncRNA Malat1 in downregulating astrocyte apoptosis and the water channel protein AQP-4 in ischemic stroke [97]. Surprisingly, almost no study of ncRNA has been discovered in the regulation of the basement membrane of the BBB in CNS diseases. The components of the basement membrane might be becoming interesting targets and deserve more research attention.

In stroke studies, sex difference has been increasingly recognized as a vital factor in determining the severity of neurological outcomes between male and female animals [220, 221]. Further efforts also need to be considered in the experimental design to investigate the roles of ncRNA in the regulation of BBB functions after cerebral ischemia.

MiRNAs have been intensively studied in the regulation of BBB in different CNS diseases, but the functional significance and molecular mechanisms of lncRNAs and circRNAs in CNS diseases are relatively less studied, especially for circRNAs, which might be due to their unique circular structure. With the development of next-generation sequencing, the advance of complete genome sequence and bioinformatics technology, more miRNAs, lncRNAs, and circRNAs will be identified, and their regulatory mechanisms on BBB permeability will also be better elucidated with the advance of methodology in neuroscience.

Despite these challenges and unsolved problems, the emerging investigations and in-depth mechanism elucidation will not only advance our current knowledge of different non-coding RNAs in the regulation of structure and function of the BBB in all CNS diseases, but also pave a fundamental basis for the development of ncRNA-based therapeutics from pre-clinical animal models to human clinical applications.

## Data Availability

Not applicable.

## Abbreviations

ncRNAs: Non-coding RNAs
CNS: Central nervous system
BBB: Blood–brain barrier
miR: MicroRNA
lncRNA: Long non-coding RNA
circRNA: Circular RNA
TBI: Traumatic brain injury
SCI: Spinal cord injury
MS: Multiple sclerosis
AD: Alzheimer's disease
VCID: Vascular cognitive impairment and dementia
SAE: Sepsis-associated encephalopathy
rRNA: Ribosomal RNA (rRNAs)
tRNA: Transfer RNAs
piRNAs: Piwi-interacting RNAs
snRNAs: Small nuclear RNAs
snoRNA: Small nucleolar RNAs
asRNAs: Antisense RNAs
PALRs: Promoter-associated long RNAs
PROMPTs: Promoter upstream transcripts
LSINCTs: Long stress-induced non-coding transcripts
3’ UTR: 3’ Untranslated region
RNAPII: RNA polymerase II
VSMC: Vascular smooth muscle cells
AJ: Adherens junction
NIHSS: National Institute of Health stroke scale
vWF: von Willebrand Factor
ICH: Intracerebral hemorrhage
PHE: Perihematomal edema
RRMS: Relapsing–remitting multiple sclerosis
EDSS: Expanded disability status scale
NF-κB: Nuclear factor-κB
ICAM-1: Intracellular adhesion molecule 1
ITGB-2: Integrin subunit beta 2
TJ: Tight junction
ZO-1: Zonula occludens 1
Aβ: Beta-amyloid
VCAM-1: Vascular cell adhesion protein 1
BCBM: Breast cancer brain metastasis
Lnc-BM: LncRNA associated with breast cancer brain metastasis
STAT3: Signal transducer and activator of transcription 3
CCL2: C–C chemokine ligand 2
MCP-1: Monocyte chemoattractant protein-1
circDLGAP4: CircRNA DLGAP4
MMP: Matrix metalloproteinase
IL: Interleukin
TNF-α: Tumor necrosis factor
MAP2k3: Mitogen-activated protein kinase kinase 3
iNOS: Inducible nitric oxide synthase
Malat1: Metastasis-associated lung adenocarcinoma transcript 1
MEG3: Maternally expressed gene 3
Snhg8: Small nucleolar RNA host gene 8
XIST: X-inactive-specific transcript
BMEC/BMVEC: Brain microvascular endothelial cell
ULK2: Uncoordinated 51-like kinase 2
AQP4: Aquaporin-4
KLF4: Krüppel-like factor 4
circDLGAP4: Circular RNA DLGAP4
EndoMT: Endothelial to mesenchymal transition
circCCDC9: Circular RNA CCDC9
eNOS: Endothelial nitric oxide synthase
ICH: Intracerebral hemorrhage
ICV: Intracerebroventricular
DUSP8: Dual-specificity phosphatase 8
HO-1: Heme oxygenase-1
SAH: Subarachnoid hemorrhage
Cav-1: Caveolin-1
VEGF: Vascular endothelial growth factor
Ang-1: Angiopoietin-1
PIK3R2: Phosphoinositide-3-kinase regulatory subunit 2
MSC: Mesenchymal stem cell
PAR-1: Protease-activated receptor-1
HDAC3: Histone deacetylase 3
Snhg3: Small nucleolar RNA host gene 3
Blnc1: Brown fat-enriched lncRNA 1
OGD: Oxygen and glucose deprivation
TWEAK: Tumor necrosis factor-like weak inducer of apoptosis
Fn14: Fibroblast growth factor-inducible 14
PPAR-γ: Peroxisome proliferator-activated receptor gamma
SIRT6: Sirtuin 6
FOXO3: Forkhead box O3
CSF: Cerebrospinal fluid
MyD88: Myeloid differentiation factor 88
PTEN: Phosphatase and tensin homolog deleted on chromosome 10
Ptch-1: Patched homolog 1
GSK3β: Glycogen synthase kinase-3β
KCNQ1OT1: KCNQ1 overlapping transcript 1
TGFβ: Transforming growth factor β
BDNF: Brain-derived neurotrophic factor
TRAF6: Tumor necrosis factor receptor-related factor 6
circLphn3: Circular RNA Lphn3
BSCB: Blood-spinal cord barrier
HMGB1: High-mobility group box-1
TLR: Toll-like receptor
ACC: Aortic cross-clamping
Mfsd2: Major facilitator superfamily domain-containing 2a
SOCS6: Suppressor of cytokine signaling 6
TUG1: Taurine upregulated gene 1
MTDH: Metadherin
BTB: Blood-tumor barrier
GECs: Glioma endothelial cells
p-PKB: Phosphorylated protein kinase B
p-Cav-1: Tyr14 phosphorylation of caveolin-1
SOX11: SRY-Box Transcription Factor 11
NGN2: Neurogenin 2
NEAT1: Nuclear paraspeckle assembly transcript 1
HOTAIR: Homeobox transcript antisense intergenic RNA
linc00174: Long intergenic non-protein coding RNA 174
CCRR: Cardiac conduction regulatory RNA
PTBP1: Polypyrimidine Tract Binding Protein 1
ZAK: ZO-1-associated kinase
cDENND4C: Circular RNA DENND4C
FLI1: Friend leukemia virus integration 1
ETV1: Ets variant gene 1
PBMC: Peripheral blood mononuclear cell
EAE: Experimental autoimmune encephalomyelitis
DOCK-1: Dedicator of cytokinesis 1
SDCBP: Syntenin-1
MMI: Multiple microinfarction
BCAS: Bilateral common carotid artery stenosis
ETS: Erythroblast transformation-specific
ERG: ETS-related gene
GM-CSF: Granulocyte–macrophage colony-stimulating factor
ELK4: ETS-domain protein 4
PbA: Plasmodium berghei ANKA
ECM: Experimental cerebral malaria
CA16: Coxsackievirus A16
Keap1: Kelch-like ECH-associated protein 1
Nrf2: Nuclear factor-erythroid factor 2-related factor 2
S1PR1: Sphingosine-1-phosphate receptor 1
NCALD: Neurocalcin delta
CLP: Cecal ligation and puncture
RCVS: Reversible cerebral vasoconstriction syndrome
Mask: Multiple Ankyrin repeats Single KH domain
TSP1: Thrombospondin-1
TSC: Tuberous sclerosis complex
TIMP: Tissue inhibitors of matrix metalloproteinases
RCVS: Reversible cerebral vasoconstriction syndrome
Npn-1: Neuropilin-1
ATG5: Autophagy Related 5
PD: Polydatin
AA: Alisol A 24-acetate
PUMA: P53 upregulated modulator of apoptosis
Cul3: Cullin 3
H_2_: Hydrogen gas
MEM: Memantine
NMDA: *N*-Methyl-d-aspartate
MAPK: Mitogen-activated protein kinase
MF: Monomethyl fumarate
CREB: CAMP-response element binding protein
PGC-1α: Peroxisome proliferator-activated receptor-gamma coactivator-1alpha
CYC C: Cytochrome *c*
S1PR2: Sphingosine-1-phosphate receptor 2
PLA2G2A: Phospholipase A2 group IIA
ALOX5: Arachidonate 5-Lipoxygenase
ITGA2B: Integrin subunit alpha 2b
JAM3: Junctional adhesion molecule 3
TJAP1: Tight junction associated protein 1
mTOR: Mammalian target of rapamycin
PI3K: Phosphoinositide 3-kinases
HMGA2: High mobility group AT-hook 2
AChE: Acetylcholinesterase
AQP11: Aquaporin-11
DUSP8: Dual specificity phosphatase 8
RUNX: Runt-related transcription factor
SOD: Superoxide dismutase
MDA: Malondialdehyde
MPO: Myeloperoxidase
SHED: Stem cells from human exfoliated deciduous teeth
CTGF: Connective tissue growth factor
MFSD2A: Major facilitator superfamily domain containing 2A
ECE1: Endothelin converting enzyme 1
SP1: Specificity protein 1
NOTCH2: Notch receptor 2
SOX: Sex determining region Y-box protein
MEF2D: Myocyte enhancer factor 2D
EMAP-II: Endothelial monocyte-activating polypeptide-II
RhoA: Ras homolog family member A
ROCKII: Rho-associated protein kinase II
PKC: Protein kinase C
MIR17HG: MiR-17-92a-1 cluster host gene
FOXR2: Forkhead box R2
PIWIL1: Piwi like RNA-mediated gene silencing 1
ITGB2: Integrin subunit beta 2
CBF: Cerebral blood flow
mBVPs: Mouse brain vascular pericytes
BMECs: Brain microvascular endothelial cells
FOXO1: Forkhead box O1
SEMA6A: Semaphorin 6A
SEMA6D: Semaphorin 6D
eef2k: Eukaryotic elongation factor 2 kinase
ceRNA: Competing endogenous RNA
Itga5: Integrin a5
